# Point-of-care ultrasound improves clinical outcomes in patients with acute onset dyspnea: a systematic review and meta-analysis

**DOI:** 10.1007/s11739-022-03126-2

**Published:** 2022-10-31

**Authors:** Gergő Vilmos Szabó, Csenge Szigetváry, László Szabó, Fanni Dembrovszky, Máté Rottler, Klemetina Ocskay, Stefanie Madzsar, Péter Hegyi, Zsolt Molnár

**Affiliations:** 1 Centre for Translational Medicine, Semmelweis University, Üllői út 26, Budapest, 1085 Hungary; 2 Emergency Department, Szent György University Teaching Hospital of Fejér County, Székesfehérvár, Hungary; 3 National Ambulance Service, Budapest, Hungary; 4 Hungarian Air Ambulance Nonprofit Ltd., Budaörs, Hungary; 5 Department of Anesthesiology and Intensive Therapy, Semmelweis University, Budapest, Hungary; 6 Institute for Translational Medicine, Medical School, University of Pécs, Pécs, Hungary; 7 Department of Anesthesiology and Intensive Therapy, Szent György University Teaching Hospital of Fejér County, Székesfehérvár, Hungary; 8 Division of Pancreatic Diseases, Heart and Vascular Centre, Semmelweis University, Budapest, Hungary; 9 Department of Anesthesiology and Intensive Therapy, Poznan University, Poznan, Poland

**Keywords:** Point-of-care ultrasound, Acute-onset dyspnea, Clinical outcomes

## Abstract

The early, appropriate management of acute onset dyspnea is important but often challenging. The aim of this study was to investigate the effects of the use of Point-of-Care Ultrasound (PoCUS) versus conventional management on clinical outcomes in patients with acute onset dyspnea. The Cochrane Library, MEDLINE, EMBASE and reference lists were searched to identify eligible trials (inception to October 14, 2021). There were no language restrictions. Randomized controlled trials (RCTs), and prospective and retrospective cohort studies that compared PoCUS with conventional diagnostic modalities (controls) in patients with acute onset dyspnea were included. Two independent reviewers extracted data and assessed the risk of bias. Disagreements were resolved by consensus. The primary study outcomes were time to diagnosis, time to treatment, and length of stay (LOS). Secondary outcomes included rate of appropriate treatment, 30-day re-admission rate, and mortality. We included eight RCTs and six observational studies with a total of 5393 participants. Heterogeneity across studies was variable (from low to considerable), with overall low or moderate study quality and low or moderate risk of bias (except one article with serious risk of bias). Time to diagnosis (mean difference [MD], − 63 min; 95% CI, − 115 to − 11 min] and time to treatment (MD, − 27 min; 95% CI − 43 to − 11 min) were significantly shorter in the PoCUS group. In-hospital LOS showed no differences between the two groups, but LOS in the Intensive Care Unit (MD, − 1.27 days; − 1.94 to − 0.61 days) was significantly shorter in the PoCUS group. Patients in the PoCUS group showed significantly higher odds of receiving appropriate therapy compared to controls (odds ratio [OR], 2.31; 95% CI, 1.61–3.32), but there was no significant effect on 30-day re-admission rate and in-hospital or 30-day mortality. Our results indicate that PoCUS use contributes to early diagnosis and better outcomes compared to conventional methods in patients admitted with acute onset dyspnea.

**Graphical abstract:**

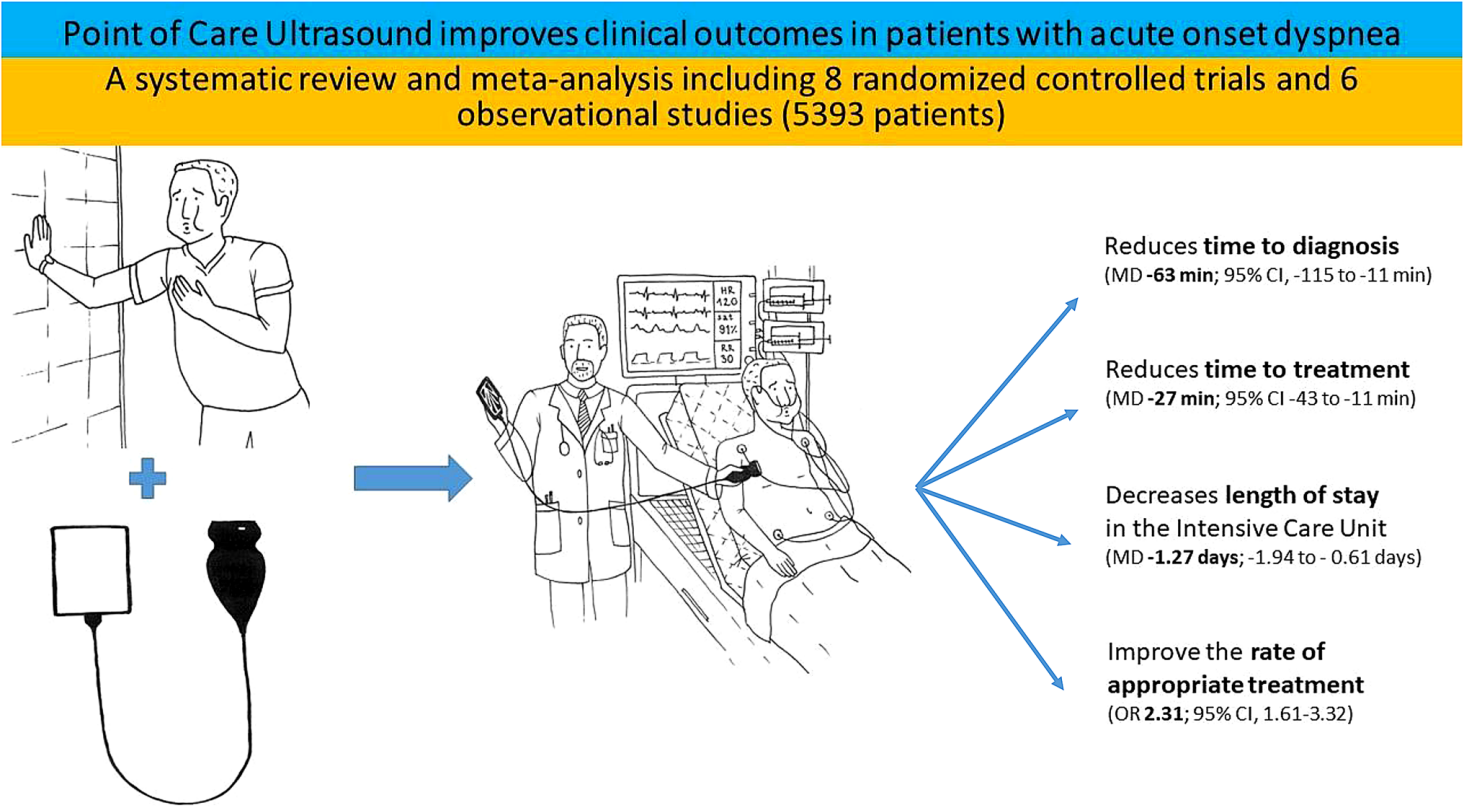

**Supplementary Information:**

The online version contains supplementary material available at 10.1007/s11739-022-03126-2.

## Introduction

Acute-onset dyspnea is one of the most common symptoms for which patients visit the Emergency Department (ED) [1–4]. In the United States, dyspnea is the main reason for four to five million ED visits annually [4], representing up to 50% of patients admitted to acute tertiary care hospitals [5]. In the Asia–Pacific region, 5% of all ED presentations are due to dyspnea [6]. In addition to its high incidence, the 30-day mortality rate of these patients remains relatively high (8–13%) [7, 8]. Therefore, rapid and appropriate diagnosis of the underlying pathology is of utmost importance for prompt and adequate treatment [9].

However, differential diagnosis is often challenging [10, 11]. Most physicians mainly rely on conventional diagnostic modalities, such as medical history, physical examination, chest X-ray (CXR), electrocardiogram (ECG), and standard laboratory tests [12]. Even given all these tests, some studies have raised doubts about the diagnostic accuracy of these conventional approaches, especially in the critically ill patient population [13, 14].

The use of Point-of-care ultrasound (PoCUS) has gained increasing popularity in several domains of acute patient management, including acute onset dyspnea [11, 15]. There is an increasing body of evidence demonstrating that the accuracy of PoCUS is comparable to the current imaging reference standard CXR in general [16] as well as in specific conditions, such as pneumonia [17], acute decompensated heart failure [16], pleural effusion [18], pneumothorax [19] and pulmonary embolism [20]. PoCUS has other advantages, such as being free from ionizing radiation, and most importantly can be performed in real-time at the bedside [16, 21]. Additionally, PoCUS can answer a broad spectrum of remaining diagnostic questions and may also help to optimize and personalize therapy [22]. However, very few trials have examined meaningful clinical outcomes related to PoCUS usage to date [23] and the results on outcome measurements were heterogeneous [24].

Therefore, we conducted a high-quality, comprehensive systematic review and meta-analysis that included the most recent publications that reported clinical outcomes with the use of PoCUS in patients who developed acute onset dyspnea. In addition to the existing diagnostic accuracy studies [16–20, 25], our main objective, as a new insight to this field, was to investigate how PoCUS improves clinical endpoints in patients with acute onset dyspnea.

## Materials and methods

### Protocol registration and search strategy

The protocol was prospectively registered via the International Prospective Register of Systematic Reviews (PROSPERO) under the registration number CRD42021284070. There was no deviation from the protocol. We report our results following the Preferred Reporting Items for Systematic Reviews and Meta-Analyses (PRISMA) recommendations [26].

We systematically searched MEDLINE (via PubMed), EMBASE, and the Cochrane Central Register of Controlled Trials (CENTRAL) for eligible articles on 14 October, 2021. We applied “title, abstract, author, keyword” filters in EMBASE—no other filters were used. We did not use any restrictions or limitations based on language or publication date. We also scanned the reference lists of included studies and the cited articles in Google Scholar. The detailed search key is outlined in the Additional Methods section.

### Selection process and data collection

Only randomized controlled trials (RCTs), and prospective and retrospective cohort studies were eligible for inclusion. Editorials, review articles, case reports, case series, conference abstracts, non-peer-reviewed articles and animal experiments were excluded.

The selected studies had to match our previously defined PICO (Patients, Intervention, Control, Outcome) framework:

P: Adults and children who were admitted to the ED or to the Intensive Care Unit (ICU), or to another inpatient setting because of acute onset or worsening dyspnea were eligible. We also included studies enrolling patients who developed shortness of breath from unknown etiologies and were already hospitalized. Studies reporting on trauma-induced acute onset dyspnea, or pregnancy were excluded.

I: The examined intervention was PoCUS use on its own or in combination with conventional diagnostic measures. If PoCUS was applied in combination with conventional methods, the endpoints in each case should be able to be evaluated separately from the control arm. There were no restrictions on the type of PoCUS protocols.

C: Control group included conventional diagnostic methods, such as taking the patients’ medical history, physical examination, ECG, blood gas and different laboratory analyses, echocardiography, CXR, or computer tomography (CT).

O: For the primary outcomes, we defined time to diagnosis (measured in minutes from admission or first medical contact until initial diagnosis was made), time to treatment (assessed as the previous point until the treatment was initiated) and length of stay (LOS) which was evaluated in the following three subgroups: in-hospital LOS, LOS in the ED and LOS in the ICU. The secondary outcomes were the following: mortality (in-hospital and 30-day), rate of appropriate treatment and 30-day re-admission rate.

After the removal of duplicates using a reference management software (EndNote X9, Clarivate Analytics), two review authors (G.S. and C.S.) independently screened titles, abstracts, and then the full texts against predefined eligibility criteria.

Cohen's kappa coefficient (κ) was calculated (by G.S. and C.S.) to measure inter-rater reliability during the selection process, where values 0.01–0.20 indicate slight, 0.21–0.40 indicate fair, 0.41–0.60 indicate moderate, 0.61–0.80 indicate substantial, and 0.81–1.00 indicate almost perfect or perfect agreement. Discrepancies were resolved by two other review authors (Z.M. and M.R.).

Based on the consensus of methodological and clinical experts, we created a standardized data collection sheet. Data on the first author, publication year, countries, study design, number of patients in each group and their baseline characteristics (including age and gender), type of PoCUS protocol, examiners’ practice and the available primary and secondary outcome parameters were extracted by two independent review authors (G.S. and C.S.) using our standardized data collection form in Microsoft Excel. There were no overlapping populations or duplicate data.

### Risk of bias and quality assessment

The risk of bias was assessed based on the recommendations of the Cochrane Collaboration. Two independent review authors (G.S. and C.S.) did the assessment, and an independent third investigator resolved any disagreements (F.D.). For RCTs the RoB 2 tool (revised tool for Risk of Bias in randomized trials) was used, whereas for the cohorts, we used the ROBINS-I tool (Risk Of Bias in Non-randomized Studies of Interventions) [27, 28].

Publication bias was assessed by visual inspection of the Funnel plots and the leave-one-out sensitivity analyses (see Additional Figs. 2 and 3).

The quality assessment of the included studies was performed with GRADE-Pro (Grading of Recommendations, Assessment, Development and Evaluation–Pro) based on the recommendations of the Cochrane Collaboration [29]. A detailed description of the quality assessment and risk of bias process can be found in the Additional Tables 1, 2, 3.

### Statistical analysis

If there were at least three studies for an outcome, a meta-analysis was performed, and the results displayed in forest plots. For continuous outcomes, pooled mean differences (MDs), and for dichotomous variables, pooled odds ratios (ORs) along with their 95% confidence interval (CI), were calculated to investigate the differences between the compared arms. A random effect model was used for meta-analyses.

If the study number for the given outcome was over five, the Hartung–Knapp adjustment [30, 31] was applied.

In all instances, raw data were used: in the case of binary data, number of event and non-event, and in the case of continuous data, mean and standard deviation (SD). If the mean and SD were not reported in the article, we estimated them from the medians, quartiles, minimum and maximums using the Luo [32] and Shi [33] methods.

To estimate the heterogeneity variance measure, *τ*^2^ was applied estimated with the Q profile method. Statistical heterogeneity across trials was assessed by means of the Cochrane *Q* test, and the *I*^*2*^ values, where *p* < 0.1 was considered as statistically significant. Due to the low number of available studies, the Egger’s test for the small-study effect could not be performed.

Outlier and influence analyses were carried out following the recommendations of Harrer et al. [34].

All statistical analyses were performed with R (R Core Team [35], v4.1.1) using the meta (Schwarzer 2022, v5.2.0) and dmetar (Cuijpers, Furukawa, and Ebert 2020, v0.0.9000) packages [36, 37].

## Results

### Search and study selection

**Fig. 1:**
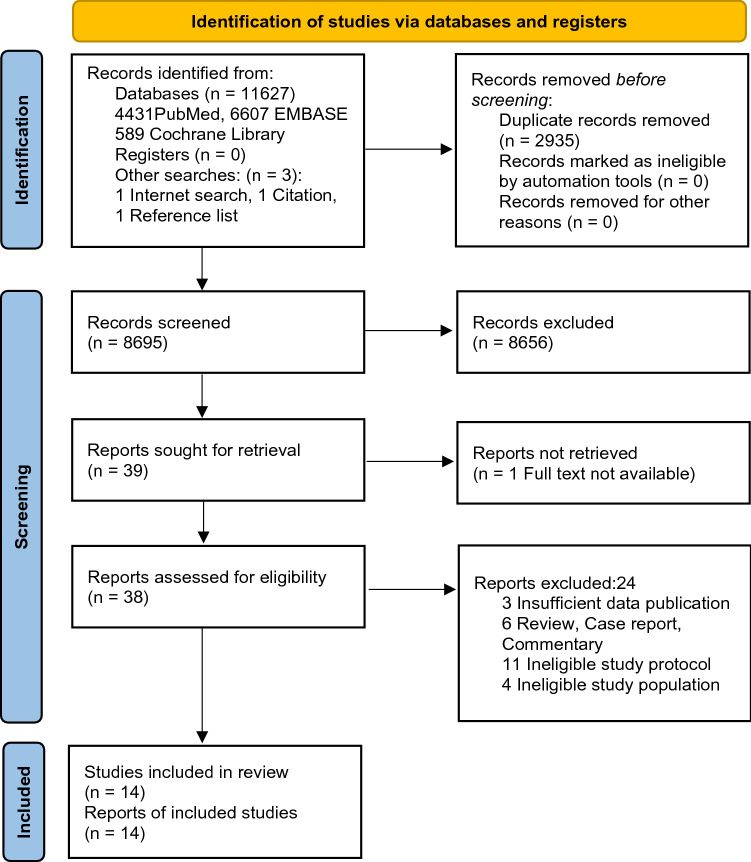
PRISMA flowchart

Based on PRISMA recommendations, the details of the electronic search are depicted in Fig. 1.

Our systematic search yielded 11,627 records and 3 other articles were found from other searches. After removing duplicates, 8695 items were screened, 32 of these were thought to be suitable for full text selection and finally 13 studies (7 RCTs [38–44] and 6 observational studies [45–50]) were processed for data collection. One additional RCT was found during an internet search which was not in the aforementioned databases [51]. Altogether 5393 patients’ data were gathered in this review, 2574 of them were female (47.7%). Cohen's kappa for abstracts and full texts was 0.67 and 0.59, respectively. The characteristics of the studies included in our systematic review and meta-analysis are presented in Table 1.

**Table 1:** Characteristic of included studies

Source	Study design (RCT /OBS)	Sample size (% male)	Examiner experience with PoCUS^a^	Examination protocol		Eligibility criteria^b,c^	Outcomes
				PoCUS protocol	Control		
Baker [40]	RCT	442 (58)	Mixed	Volpicelli’s 8 view, subcostal cardiac clip (posterior lung not tested)	Medical history, physical examination, ECG, blood test, CXR, echocardiography, CT	Inc: ≥ 60 years, able to understand and sign a written consent, not requiring immediate resuscitation Exc: no data	Length of stay, mortality
Blans [46]	OBS	61^d^ (52)	Beginner	BLUE, cardiac: standard transthoracic windows: LV/RV dilatation and function, pericardial tamponade / effusion, subcostal view: IVC	Not stated	Inc: call for MET based on Modified Early Warning Score Exc: pregnancy, requiring direct lifesaving intervention, GCS < 9 or GCS declined ≥ 2 as the primary reason for MET attendance	Mortality
Colclough [38]	RCT	40 (55)	Not specified	Cardiac (based on Preoperative Pocket Echocardiography Trial)	Not stated	Inc: National Health Service triage category 1–3 Exc: no data	Time to diagnosis, mortality
Corsini [47]	OBS	124 (61)	Beginner	Bilateral anterior, Lateral, and posterior lung ultrasound, transabdominal scanning for lung bases and subcostal for diaphragm	CXR	Inc: ≥ 23 week of gestational age, RR > 60, oxygen supplementation, respiratory support Exc: CPR	Time to diagnosis
Harel [48]	OBS	202 (61)	Not specified	no data	CXR	Inc: < 18 years, suspected pneumonia Exc: ED left before discharge, both PoCUS and CXR were made, PoCUS undertaken not by patient’s treating physician	Length of stay, re-admission rate
Laursen [39]	RCT	315 (43)	Expert	FATE protocol, modified Volpicelli’s 8 view, deep veins according to American College of Emergency Medicine’s criteria	Blood samples, blood gasses, ECG, CXR, CT, echocardiography	Inc: RR > 20, SAT < 95%, coughing, chest pain Exc: permanent mental disability, PoCUS not done within 1 h after the primary assessment	Length of stay, re-admission rate, mortality
Nakao [45]	OBS	324 (49)	Not specified	Volpicelli’s 8 view	Not stated	Inc: ≥ 50 years, suspected acute heart failure or COPD exacerbation Exc: ST-elevation myocardial infarction, known interstitial fibrosis, lobectomy or PTX	Time to treatment, length of stay
Pivetta [41]	RCT	518 (53)	Not specified	Volpicelli’s 8 view	Past medical history, history of present illness, physical examination, arterial blood gas analysis, ECG, CXR, N-terminal pro-brain natriuretic peptide	Inc: sudden onset of dyspnea or increase in the severity of chronic dyspnea in the previous 48 h Exc: mechanically ventilated at the time of first evaluation, dyspnea in context of trauma	Time to diagnosis, length of stay, mortality
Riishede [42]	RCT	211 (51)	Expert	Volpicelli’s 8 view (modified), subcostal or apical cardiac (4-chamber: pericardial effusion, LV function, RV overload)	clinical examination, blood samples, ECG, CXR, CT, echocardiography	Inc: coughing, chest pain, RR > 20, SAT < 95% Exc: PoCUS already done, inability to randomize or do PoCUS < 4 h	Appropriate treatment, re-admission rate, mortality
Seyedhosseini [43]	RCT	50 (58)	Mixed	BLUE protocol	Patients’ history, physical examination, CXR, biochemistry, CT	Inc: > 12 years, Acute Respiratory Distress Syndrome within the past 7 days Exc: dyspnea due to previously diagnosed medical condition, need CPR on arrival	Time to treatment, length of stay, mortality
Wang [44]	RCT	128 (51)	Expert	BLUE protocol, parasternal long-axis view to assess cardiac contractility and left ventricular ejection fraction, subxiphoid view to assess IVC	Bedside CXR, central venous and arterial blood gas parameters, myocardial injury marker levels, pulse index contour continuous cardiac output catheter, pulmonary artery catheter	Inc: admitted to ICU with acute pulmonary edema, dyspnea in 48 h, partial arterial oxygen pressure / fraction of inspired oxygen < 300 mmHg, bedside CXR showing ≥ 1 new sign of acute pulmonary edema according to the assessment of the attending ICU physician Exc: history of chronic cardiac dysfunction	Time to diagnosis, length of stay, mortality
Wang [51]	RCT	130 (49)	Expert	Extended FATE and BLUE-plus protocols were modified into a critical care ultrasonic examination protocol	Vital signs, medical history, physical examination, laboratory tests, CXR, CT	Inc: required emergent critical consultation for pulmonary or circulation failures from medical / surgical units, post-surgical patients Exc: refused ICU transfer, already experienced cardiac arrest, advanced cancer	Time to diagnosis, time to treatment, mortality
Zanobetti [49]	OBS	2683 (51)	Expert	LUS (longitudinal and oblique scans on anterolateral and posterior thoracic areas, according to Volpicelli), cardiac (apical 4-chamber view to evaluate left ventricular ejection fraction or presence of right ventricular dilatation, subcostal long axis to assess pericardial effusion and left ventricular ejection fraction), IVC	Vital signs, medical history, physical examination, ECG, CXR, CT, echocardiography, blood sampling or arterial blood gas	Inc: acute dyspnea of every degree Exc: traumatic origin, discharged after ED evaluation	Time to diagnosis
Zieleskiewicz [50]	OBS	165 (62)	Mixed	Cardiac (left and right ventricular function, pulmonary assessment), BLUE protocol, imaging of the deep veins when deemed necessary	Taking medical history, performance of a circulatory, respiratory and neurological assessment, vital signs, blood testing, conduction of any additional tests judged necessary by the physician	Inc: medical or surgical wards and developing respiratory and/or circulatory failure justifying placement of a call to the RRT Exc: pregnancy, cardiac arrest, technical limitations to the performance of US, lung or cardiac transplant, RRT call for a neurological failure, RRT call by the ED and impossible follow-up	Time to diagnosis, time to treatment, length of stay, appropriate treatment, mortality

*BLUE* Bedside Lung Ultrasound in Emergency, *CPR* cardiopulmonary resuscitation, *Exc* exclusion, *FATE* Focus Assessed Transthoracic Echocardiography, *GCS* Glasgow Coma Scale, *Inc* inclusion, *IVC* inferior vena cava (diameter), *LUS* lung ultrasound, *LV* left ventricule, *MET* Medical Emergency Team, *OBS* observational study, *RCT* Randomized Control Trial, *RR* respiratory rate/min, *RRT* rapid response team, *RV* right ventricule, *SAT* peripheral oxygen saturation

^a^Examiner practice: beginner: trained in basic level and/or low clinical experience; expert: trained in high level and/or high level of clinical experience

^b^Consent and dyspnea as an eligibility criteria is not specifically mentioned, due to being omnipresent

^c^Age restriction is highlighted only when children or older population were included

^d^We received data from the authors just about patients treated with respiratory failure

### Primary outcomes

**Fig. 2:**
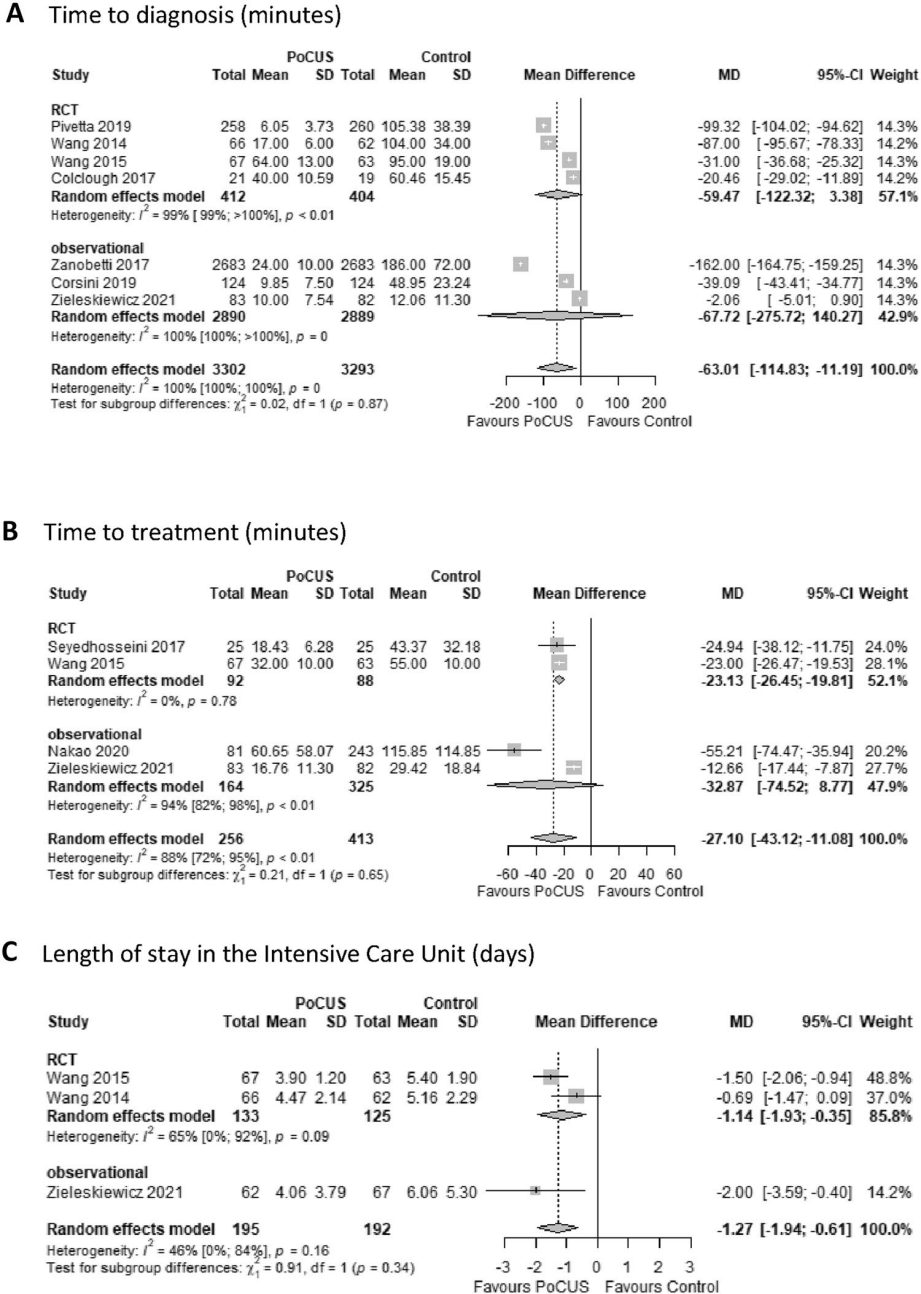
Primary outcomes in patients admitted with acute onset dyspnea when PoCUS was used compared to conventional modalities (control). Comparison of patients admitted with dyspnea examined by PoCUS vs conventional modalities in time to diagnosis (considerable heterogeneity detected) (**A**), time to treatment (considerable heterogeneity detected) (**B**), and length of stay in the Intensive Care Unit (moderate heterogeneity detected) (**C**). PoCUS indicates Point of Care Ultrasound; SD, standard deviation; MD, mean difference. The size of squares is proportional to the weight of each study. Horizontal lines indicate the 95% CI of each study; diamond, the pooled estimate with 95% CI

Time to diagnosis was the most cited endpoint in the studies (7 of 15). PoCUS use compared to controls resulted in a significant reduction in time to making the diagnosis (MD − 63 min; 95% CI − 115 to − 11 min) (Fig. 2A). Time to treatment was reported in four studies. In the PoCUS group, patients also received treatment significantly earlier (MD − 27 min; 95% CI − 43 to − 11 min) compared to controls (Fig. 2B). Heterogeneity among these trials for both outcomes was considerable (*I*^*2*^ = 100%, *p* = 0 and 88%, *p* < 0.01, respectively).

As far as in-hospital LOS is concerned, PoCUS use showed no significant effect (MD − 0.02 days; 95% CI − 0.43 to 0.39 days), with low heterogeneity (*I*^*2*^ = 0%, *p* = 0.81). Regarding LOS in the ED, there was a mean of 35 min less waiting time to discharge or admission to a ward that proved not significant (MD − 35 min; 95% CI − 93 to 23 min), but heterogeneity was high (*I*^*2*^ = 84%, *p* < 0.01). Patients in the PoCUS group stayed for a significantly shorter time in the ICU than controls (MD − 1.27 days; 95% CI − 1.94 to − 0.61 days) (Fig. 2C). Heterogeneity was moderate among these trials (*I*^*2*^ = 46%, *p* = 0.16).

### Secondary outcomes

**Fig. 3:**
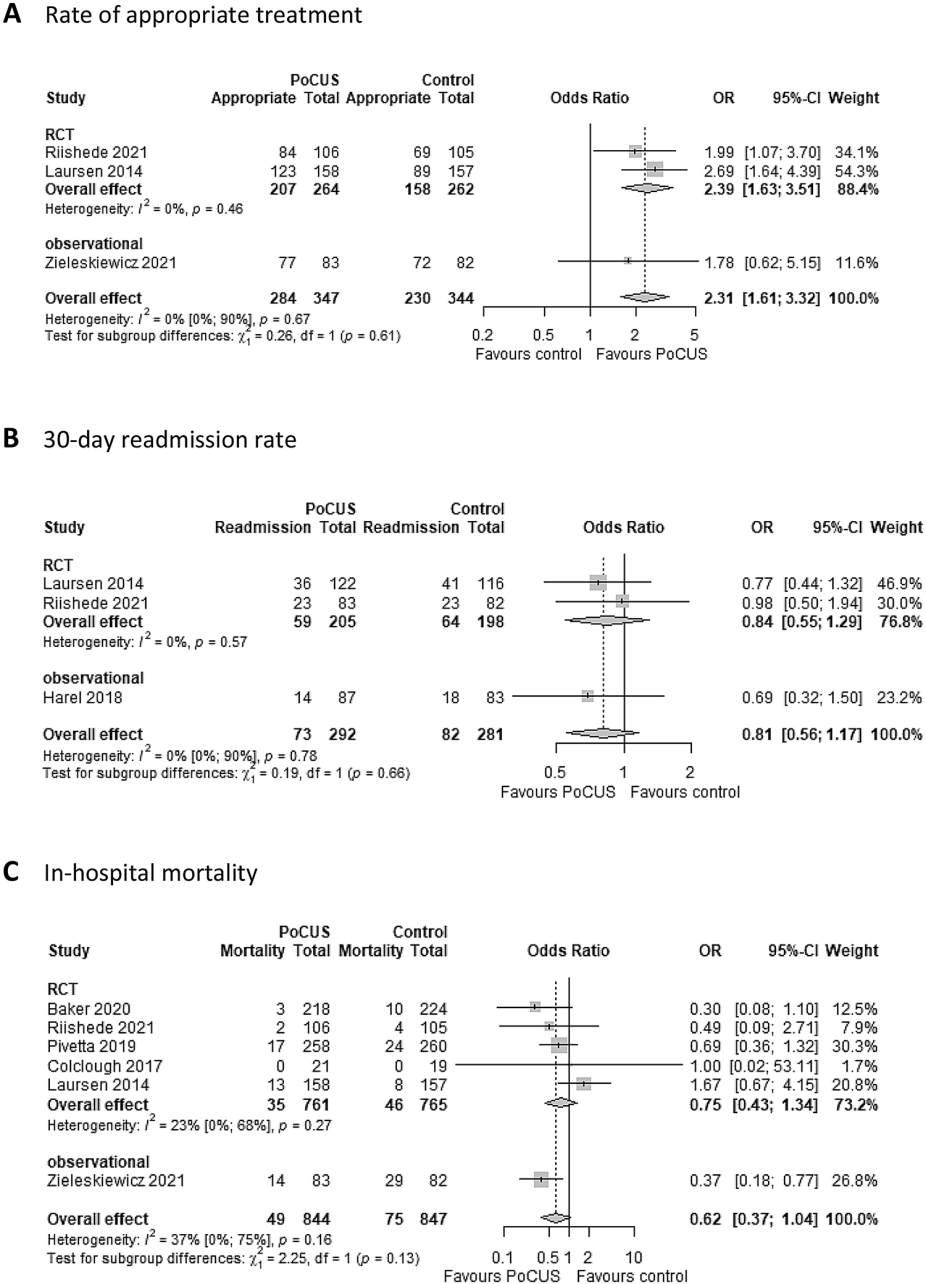
Secondary outcomes in patients admitted with dyspnea when PoCUS was used compared to conventional modalities (control). Comparison of patients admitted with dyspnea examined by PoCUS vs conventional modalities in rate of appropriate treatment (low heterogeneity detected) (**A**), 30-day re-admission rate (low heterogeneity detected) (**B**), and in-hospital mortality (moderate heterogeneity detected) (**C**). PoCUS indicates Point of Care Ultrasound; OR, odds ratio. The size of squares is proportional to the weight of each study. Horizontal lines indicate the 95% CI of each study; diamond, the pooled estimate with 95% CI

Regarding secondary endpoints, patients in the PoCUS group had significantly higher odds (OR 2.31; 95% CI 1.61 to 3.32) of receiving appropriate therapy compared to controls, and studies showed low heterogeneity (*I*^*2*^ = 0%, *p* = 0.67) (Fig. 3A).

We found no significant effects on 30-day re-admission rate (OR, 0.81; 95% CI, 0.56 to 1.17) with low heterogeneity (*I*^*2*^ = 0%, *p* = 0.78); 30-day mortality (OR, 0.82; 95% CI 0.31–2.18) and in-hospital mortality (OR 0.62; 95% CI 0.37 to 1.04), with moderate heterogeneity (*I*^*2*^ = 50%, *p* = 0.11 and *I*^*2*^ = 37%, *p* = 0.16, respectively) (Fig. 3 and Additional Fig. 1). However, in the latter outcome, one article (Laursen [39]) appeared to be a potential outlier, but due to the low number of studies, the leave-one-out-analysis was discussed only in the Additional file (for more details see Additional Fig. 1).

### Risk of bias assessment, publication bias and certainty of evidence

Based on the Cochrane proposal, the risk of bias assessment showed serious concern for only one article [48] and moderate (some concern in cases of RCTs) or low risk for all others. For GRADE, the certainty of evidence in the studies was variable, only the rate of appropriate treatment fell into high certainty category. The results of the risk of bias assessment of individual studies, the Funnel plots and the leave-one-out sensitivity analyses are shown in the Additional Files (Additional Tables 2, 3 and Additional Figs. 2, 3). Furthermore, the final GRADE assessment is also shown in Additional Table 1.

## Discussion

### Statement of principal findings

The results of this systematic review and meta-analysis have shown that patients admitted with acute onset dyspnea and managed with PoCUS have a significantly shorter time to diagnosis, time to treatment, higher rate of receiving appropriate treatment, and decreased stay in ICU compared to conventional approaches. However, use of PoCUS has a limited influence on 30-day and in-hospital mortality and had no relevant effect on the 30-day re-admission rate.

Due to the fact that approximately 20% of patients presenting to the ED with dyspnea are misdiagnosed and consequently inappropriately treated [52], PoCUS could have a potential role as an important diagnostic tool in patient management [53]. Our results provide high-level evidence to support this hypothesis. PoCUS has several advantages over conventional modalities, such as immediate availability of results [16], lack of ionizing radiation [21], cost-effectiveness [54], reproducibility [11], independency of the patients’ breath-holding capacity [11], portability and safety [55]. Although PoCUS use has increased substantially in critical care settings over the last two decades [11, 56, 57], it still remains underused [19], as indicated by the lower-than-expected prevalence of PoCUS devices in rural areas [58] and its use in only around 5% of patients in the ED [59]. This tendency can, in part, be explained by the lack of standardized training facilities [21], the operator dependency that hinders quality assurance [60], and most importantly the lack of high-quality evidence-based guidelines on PoCUS [55, 57, 61]. Our results provide substantial evidence that PoCUS use should be promoted on both national and international levels, and measures should be taken to improve its implementation and practice.

### Strengths and weaknesses of the study

To the best of our knowledge, this systematic review and meta-analysis on the use of PoCUS in patients with acute onset dyspnea is one of the largest and most comprehensive studies to date. The strengths are the application of a rigorously followed protocol prospectively registered on PROSPERO, the evaluation of the overall quality of evidence using the GRADE system, and being up to date by incorporating the most recent literature. We also included studies examining clinical outcomes, regardless of their language or publication date, not just those evaluating diagnostic accuracy. Additional strengths include the assessment of highly relevant clinical outcomes [53] and the fact that there were no relevant missing data in the included studies. In contrast to previous reviews and meta-analyses [16–20, 60] that analyzed data from patients with an explicit diagnosis, such as pneumonia or acute decompensated heart failure [9, 10, 16, 17, 62], we applied a broader definition of dyspnea, thereby including more patients and providing more comprehensive results.

In the case of one study (Blans [46]), the author kindly provided the original data on patients with dyspnea, excluding all other causes. This allowed us to have a more homogeneous population and is the reason for the differences in patient numbers presented in their original article and in our analyses.

Our study also has certain limitations. There was substantial heterogeneity regarding the age groups as we included infants and patients older than 59 years [40, 47]. Severity of illness, as indicated by the patients’ different medical conditions, also showed heterogeneity as some articles included intubated, mechanically ventilated patients, while others excluded this group [41, 47]. Furthermore, not all patients had dyspnea only as the sole complaint. Some articles also included patients with coughing or chest pain, which further increased the heterogeneity of the study population (Table 1). However, we tried to overcome this issue by including studies where the majority of subjects required medical intervention for acute onset dyspnea and included them in data collection and analysis. The diversity of PoCUS protocols may be another important factor behind the high heterogeneity of the results and this is a key point and limitation at the same time, from both the methodological and clinical points of view. For example, some studies used PoCUS only to investigate the lungs, whereas others examined the heart or both heart and lungs, while some studies also evaluated the venous system (Table 1). Furthermore, there is a lack of standardization regarding PoCUS training and practice. Hence, we cannot exclude that in this regard there was substantial diversity in the included studies.

Additionally, there were also some challenges in the interpretation of the reported data. For example, extracting numerical data from figures was particularly difficult, and in one case [48] the re-admission rate period was 21 days instead of 30 days.

Regarding the outcomes, on the one hand, it should be noted that time to diagnosis could be influenced by the operator’s experience. On the other hand, classification of the primary and secondary end points was arbitrarily defined by us at the time of the PROSPERO registration. This was followed throughout the analysis and not modified subsequently, although not all articles used exactly the same classification as we did.

Nevertheless, these limitations highlight the importance and need for the development of gold standards for the management of this patient population to improve quality of care.

### Comparison with other studies

Several reviews have investigated the diagnostic accuracy of PoCUS in patients with dyspnea [9, 10, 17, 18, 20, 24, 25, 60, 62], but only a couple have included similar outcomes to ours [19, 63].

Our results are in contrast with a recently published guideline [61], which states that clinicians may use PoCUS in addition to the standard diagnostic pathway when there is diagnostic uncertainty. Based on our results, we recommend that all patients suffering from acute onset dyspnea should be managed by PoCUS as a standard and not only as a supplementary tool when standard diagnostic measures fail.

Alrajab et al. [19] reported in a meta-analysis that the PoCUS group needed a significantly shorter time to show the presence or absence of pneumothorax. Their results are in line with our findings that PoCUS use can reduce time to diagnosis by more than one hour. A recent systematic review and meta-analysis that included 49 studies with data on 9782 participants found that PoCUS had no effect on in-hospital LOS [63], which is in accordance with our results. To the best of our knowledge, ED and ICU staying as separate outcomes have not been evaluated in previous meta-analyses. Hence, ours is the first to report on these. According to our results, PoCUS use may reduce LOS in ED and the ICU, which could have other potential beneficial effects (e.g., decreased costs and/or reduced emergency room wait times) that should be investigated in future.

Regarding 30-day re-admission rates, although there was a tendency in favor of PoCUS, similar to the American College of Physicians guideline [61], we could not demonstrate any statistically significant effect.

Garthlehner et al. [63] found no statistically significant differences for in-hospital mortality based on the analysis of three RCTs [39–41]. Since this review, two further studies have been published [42, 50] that were included in our analysis, thereby we found a tendency toward PoCUS reducing in-hospital mortality, but it was not significant. Nevertheless, this positive signal in our study should encourage further research in the field.

In a prospective, comparative study by Silva et al. [64], PoCUS, compared to routine clinical assessment, significantly improved the rate of appropriate treatment in patients admitted to ICU with acute respiratory failure. However, it is important to note that this outcome was defined based on local treatment guidelines which may differ from center to center, and in one article [50] was not defined at all. In our analysis, we included patients from the ED as well as from medical and surgical wards. Our results from a broader perspective also suggest that the rate of appropriate treatment can definitely be improved using PoCUS.

### Implications for practice

The results of this systematic review and meta-analysis indicate that all patients admitted with acute onset dyspnea should be examined with PoCUS to reduce time to diagnosis, time to treatment, LOS and potentially mortality.

### Implication for research

There are several positive signals in our results that should encourage further research in this field. To optimize PoCUS use in daily routine, further studies are needed in which patient selection criteria provide a more homogeneous population, and the experience of the examiners is also well defined. Finally, standardizing PoCUS protocols is of paramount importance and is a challenging task for the future.

## Conclusion

The results of this systematic review and meta-analysis support the use of PoCUS to improve differential diagnosis, achieve early appropriate treatment and decrease LOS in the ICU compared to conventional diagnostic modalities in patients admitted with acute onset dyspnea.

## Supplementary Information

Below is the link to the electronic supplementary material.

Supplementary file1 (DOCX 386 KB)
