# Supplementary material for: Point-of-care ultrasound improves clinical outcomes in patients with acute onset dyspnea: a systematic review and meta-analysis
Source: Intern Emerg Med. 2022 Oct 31;18(2):639–53. doi: 10.1007/s11739-022-03126-2 (PMC10017566; doi:10.1007/s11739-022-03126-2)
Supplement: Supplementary file 1 — Supplementary file1 (DOCX 386 KB) [file 11739_2022_3126_MOESM1_ESM.docx]

**Additional Files – Supplementary Online Content**

**Gergő VSz, Csenge Sz, László Sz et al. Point of Care Ultrasound improves clinical outcomes in patients with acute onset dyspnea—a systematic review and meta-analysis**

**Additional Table 1.** Grading of Recommendations, Assessment, Development and Evaluation (GRADE) method

**Additional Table 2.** Assessments of the risks of bias in eligible studies according to the Revised Cochrane risk-of-bias tool for randomized trials

**Additional Table 3.** Assessments of the risks of bias in eligible studies according to the Risk Of Bias In Non-randomized Studies of Interventions (ROBINS-I) assessment tool

**Additional Figure 1.** Outcomes in patients admitted with acute onset dyspnea when PoCUS was used compared to conventional modalities (control)

**Additional Figure 2.** Funnel Plots of the Meta-Analysis

**Additional Figure 3.** The leave-one-out sensitivity analyses

**Additional Method** Search key

**Additional Table 1: Grading of Recommendations, Assessment, Development and Evaluation (GRADE) method**

| **Certainty assessment** | | | | | | | **№ of patients** | | **Effect** | | **Certainty** | **Importance** |
| --- | --- | --- | --- | --- | --- | --- | --- | --- | --- | --- | --- | --- |
| **№ of studies** | **Study design** | **Risk of bias** | **Inconsistency** | **Indirectness** | **Imprecision** | **Other considerations** | **PoCUS** | **conventional modalities** | **Relative (95% CI)** | **Absolute (95% CI)** |  |  |
| **Rate of appropriate treatment** | | | | | | | | | | | | |
| 2 | randomised trials | not serious | not serious | serious^a^ | not serious | strong association | 207/264 (78.4%) | 158/262 (60.3%) | **OR 2.39** (1.63 to 3.51) | **181 more per 1 000** (from 109 more to 239 more) | ⨁⨁⨁⨁ High | CRITICAL |
| **In-hospital mortality** | | | | | | | | | | | | |
| 5 | randomised trials | not serious | serious^b^ | serious^c^ | serious^d^ | none | 35/761 (4.6%) | 46/765 (6.0%) | **OR 0.75** (0.43 to 1.34) | **14 fewer per 1 000** (from 33 fewer to 19 more) | ⨁◯◯◯ Very low | IMPORTANT |
| **30-day mortality** | | | | | | | | | | | | |
| 3 | randomised trials | not serious | serious^e^ | serious^f^ | serious^g^ | none | 21/285 (7.4%) | 20/281 (7.1%) | **OR 0.65** (0.15 to 2.73) | **24 fewer per 1 000** (from 60 fewer to 102 more) | ⨁◯◯◯ Very low | IMPORTANT |
| **Time to diagnosis** | | | | | | | | | | | | |
| 4 | randomised trials | serious^h^ | serious^i^ | serious^j^ | not serious | strong association | 412 | 404 | - | MD **59 minutes fewer** (122 fewer to 3 more) | ⨁⨁◯◯ Low | CRITICAL |
| **Time to diagnosis** | | | | | | | | | | | | |
| 3 | observational studies | not serious | serious^k^ | serious^l^ | not serious | strong association | 2890 | 2889 | - | MD **68 minutes fewer** (276 fewer to 140 more) | ⨁⨁⨁◯ Moderate | CRITICAL |
| **30-day readmission rate** | | | | | | | | | | | | |
| 2 | randomised trials | not serious | not serious | serious^m^ | not serious | none | 59/205 (28.8%) | 64/198 (32.3%) | **OR 0.84** (0.55 to 1.29) | **37 fewer per 1 000** (from 115 fewer to 58 more) | ⨁⨁⨁◯ Moderate | IMPORTANT |
| **Time to treatment** | | | | | | | | | | | | |
| 2 | randomised trials | serious^n^ | serious^o^ | serious^p^ | not serious | strong association | 92 | 88 | - | MD **23 minutes lower** (26 lower to 20 higher) | ⨁⨁◯◯ Low | CRITICAL |
| **Time to treaement** | | | | | | | | | | | | |
| 2 | observational studies | not serious | serious^q^ | serious^r^ | not serious | strong association | 164 | 325 | - | MD **33 minutes lower** (75 lower to 9 higher) | ⨁⨁⨁◯ Moderate |  |
| **Length of stay (Emergency Department)** | | | | | | | | | | | | |
| 2 | observational studies | very serious^s^ | serious^t^ | serious^u^ | not serious | strong association | 182 | 344 | - | MD **63 minutes lower** (97 lower to 29 lower) | ⨁◯◯◯ Very low |  |
| **Length of stay (in-hospital)** | | | | | | | | | | | | |
| 5 | randomised trials | not serious | serious^v^ | serious^w^ | not serious | none | 709 | 710 | - | MD **0.04 days lower** (0.5 lower to 0.43 higher) | ⨁⨁◯◯ Low | IMPORTANT |
| **Length of stay (Intensive Care Unit)** | | | | | | | | | | | | |
| 2 | randomised trials | serious^x^ | serious^y^ | serious^z^ | not serious | very strong association | 133 | 125 | - | MD **1.14 days lower** (1.93 lower to 0.35 lower) | ⨁⨁⨁◯ Moderate | CRITICAL |

**CI:** confidence interval; **MD:** mean difference; **OR:** odds ratio

**Explanations**

a. Chest pain or coughing alone was enough for inclusion even without dyspnea. (Laursen and Riishede)

b. Sicker patients were incorporated: just TRIAGE I-III, IV-V not (Colclough). Older population: >59y (Baker). Laursen go to other direction than others.

c. Chest pain or coughing alone was enough for inclusion (Riishede). Chest pain or coughing alone was enough for inclusion (Laursen).

d. It was a rare event: 81/1526= 5%

e. Laursen found the opposite result as the others. Sicker patients were incorporated: just TRIAGE I-III, IV-V not (Colclough). I2 = 67 %

f. Chest pain or coughing alone was enough for inclusion even without dyspnea. (Laursen and Riishede)

g. It was a rare event: 41/566 = 7 %.

h. For details see Risk of Bias table.

i. Inconsistency: I2: 99 %. Different populations in the studies (ICU, ER, RRT in the ward): sicker patients were incorporated: just TRIAGE I-III, IV-V not (Colclough), sicker patients: need ICU consultation (MET), APACHE II score: 13 points (Wang 2015), sicker patients:ICU patients: PaO2/FiO2<300 mm Hg (Wang 2014).

j. Circulation failures were included and some patients were treated due to shock not to dyspnea alone (Wang 2015). And to other consideration: I think it was a large effect, that 59 min time reduction could be achieved, so I have upgraded for this.

k. Inconsistency: I2: 100 %. Different populations in the studies (PICU, ER, RRT/MET in the ward). Younger population: infants (Corsini). Sicker population, because MET intervention was necessary (so mortality was also higher) - Zieleskiewicz.

l. Just 70 % of the patients' were mainly treated with dyspnea; circulatory failure was also suitable for inclusion (Zieleskiewicz). And to other consideration: I think it was a large effect, that 68 min time reduction could be achieved, so I have upgraded for this.

m. Chest pain or coughing alone was enough for inclusion (Laursen). Chest pain or coughing alone was enough for inclusion (Riishede).

n. ROB 2 showed some concerns in both studies.

o. Sicker patients: need ICU consultation (MET), APACHE II score: 13 points (Wang 2015).

p. Circulation failures were included and some patients were treated due to shock not to dyspnea alone (Wang 2015). And to other consideration: I think it was a large effect, that 23 min time reduction could be achieved, so I have upgraded for this.

q. Sicker population, because MET intervention was necessary (so mortality was also higher) - Zieleskiewicz. Older population (age >49y) - Nakao.

r. Only patients were included who has suspected COPD or ADHF and coughing alone was enough (perhaps without dyspnea) - Nakao. Just 70 % of the patients' were mainly treated with dyspnea; circulatory failure was also suitable for inclusion (Zieleskiewicz). And to other consideration: I think it was a large effect, that 33 min time reduction could be achieved, so I have upgraded for this.

s. Harel: ROBINI-I domain 3. There is a serious risk of bias in classification of interventions based on ROBINS-I algorithm. There is no explanation why that 101 patients RTG results were observed from the 5606 suitable.

t. Older population (age >49y) - Nakao. Only children were included (<18y) - Harel.

u. Only patients were included who has suspected COPD or ADHF and coughing alone was enough (perhaps without dyspnea) - Nakao. Just patients with pneumonia were included - some kind of bias (Harel). And to other consideration: I think it was a large effect, that 63 min time reduction could be achieved, so I have upgraded for this.

v. Sicker patients:ICU patients: PaO2/FiO2<300 mm Hg (Wang 2014). Older population: >59y (Baker).

w. Chest pain or coughing alone was enough for inclusion (Laursen).

x. Both studies showed some concern in ROB 2. I2 = 65 %.

y. Sicker patients: need ICU consultation (MET), APACHE II score: 13 points (Wang 2015). Sicker patients:ICU patients: PaO2/FiO2<300 mm Hg (Wang 2014).

z. Circulation failures were included and some patients were treated due to shock not to dyspnea alone (Wang 2015) And to other consideration: I think it was a large effect, that 1.14 day min time reduction could be achieved, so I have upgraded for this with 2 points.

**Additional Table 2: Assessments of the risks of bias in eligible studies according to the Revised Cochrane risk-of-bias tool for randomized trials**

| Study | Outcome | Randomization process | Deviations from intended interventions | Missing outcome data | Measurement of the outcome | Selection of the reported result | Overall |
| --- | --- | --- | --- | --- | --- | --- | --- |
| Baker, 2020 | length of stay | low | low | low | low | low | low |
| Baker, 2020 | mortality | low | low | low | low | low | low |
| Colclough, 2017 | time to diagnosis | some concerns | low | low | low | low | some concerns |
| Colclough, 2017 | mortality | some concerns | low | low | low | low | some concerns |
| Laursen, 2014 | length of stay | low | low | low | low | low | low |
| Laursen, 2014 | appropriate treatment | low | low | low | low | low | low |
| Laursen, 2014 | readmission rate | low | low | low | low | low | low |
| Laursen, 2014 | mortality | low | low | low | low | low | low |
| Pivetta, 2019 | time to diagnosis | low | low | low | low | low | low |
| Pivetta, 2019 | length of stay | low | low | low | low | low | low |
| Pivetta, 2019 | mortality | low | low | low | low | low | low |
| Riishede, 2021 | appropriate treatment | low | low | low | low | low | low |
| Riishede, 2021 | readmission | low | low | low | low | low | low |
| Riishede, 2021 | mortality | low | low | low | low | low | low |
| Study | Outcome | Randomization process | Deviations from intended interventions | Missing outcome data | Measurement of the outcome | Selection of the reported result | Overall |
| Seyedhosseini, 2017 | time to diagnosis | low | low | low | low | some concerns | some concerns |
| Seyedhosseini, 2017 | time to treatment | low | low | low | low | some concerns | some concerns |
| Seyedhosseini, 2017 | mortality | low | low | low | low | some concerns | some concerns |
| Wang, 2014 | time to diagnosis | some concerns | low | low | low | some concerns | some concerns |
| Wang, 2014 | length of stay | some concerns | low | low | low | some concerns | some concerns |
| Wang, 2014 | mortality | some concerns | low | low | low | some concerns | some concerns |
| Wang, 2015 | time to diagnosis | some concerns | low | low | low | some concerns | some concerns |
| Wang, 2015 | time to treatment | some concerns | low | low | low | some concerns | some concerns |
| Wang, 2015 | length of stay | some concerns | low | low | low | some concerns | some concerns |

**Additional Table 3: Assessments of the risks of bias in eligible studies according to the Risk Of Bias In Non-randomized Studies of Interventions (ROBINS-I) assessment tool**

| **Study** | **Outcome** | **Signalling questions** | **Bias in selection of participants into the study** | **Bias in classification of interventions** | **Bias due to deviations from intended interventions** | **Bias due to missing data** | **Bias in measurement of outcomes** | **Bias in selection of the reported result** | **Overall bias** |
| --- | --- | --- | --- | --- | --- | --- | --- | --- | --- |
| Blans, 2021 | mortality | low | low | low | low | low | low | low | low |
| Corsini. 2019 | time to diagnosis | low | low | low | low | low | low | low | low |
| Harel, 2018 | length of stay | low | low | serious | low | low | low | low | serious |
| Harel, 2018 | readmission | low | low | serious | low | low | low | low | serious |
| Nakao, 2020 | time to diagnosis | low | low | low | low | low | low | low | low |
| Nakao, 2020 | length of stay | low | low | low | low | low | low | low | low |
| Zanobetti, 2017 | time to diagnosis | low | low | low | low | low | low | low | low |
| Zieleskiewicz, 2021 | time to diagnosis | low | low | low | low | low | moderate | low | moderate |
| Zieleskiewicz, 2021 | time to treatment | low | low | low | low | low | moderate | low | moderate |
| Zieleskiewicz, 2021 | length of stay | low | low | low | low | low | low | low | low |
| Zieleskiewicz, 2021 | appropriate treatment | low | low | low | low | low | low | low | low |
| **Study** | **Outcome** | **Signalling questions** | **Bias in selection of participants into the study** | **Bias in classification of interventions** | **Bias due to deviations from intended interventions** | **Bias due to missing data** | **Bias in measurement of outcomes** | **Bias in selection of the reported result** | **Overall bias** |
| Zieleskiewicz, 2021 | mortality | low | low | low | low | low | low | low | low |

**Additional Figure 1: Outcomes in patients admitted with acute onset dyspnea when PoCUS was used compared to conventional modalities (control)**


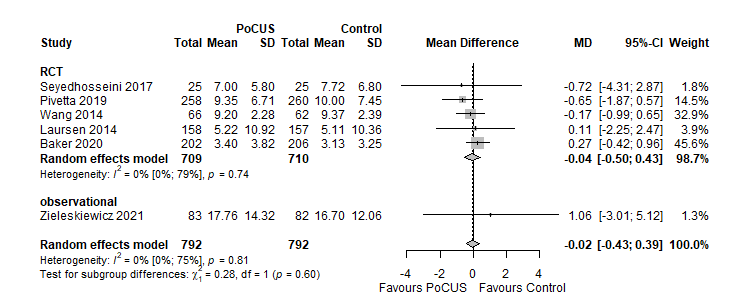

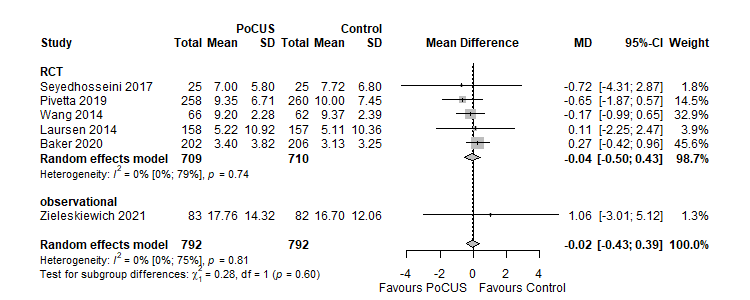
**A** Length of stay – in-hospital (days)


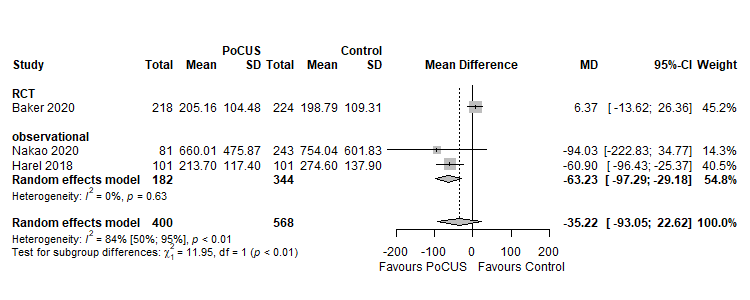
**B** Length of stay – Emergency Department (minutes)


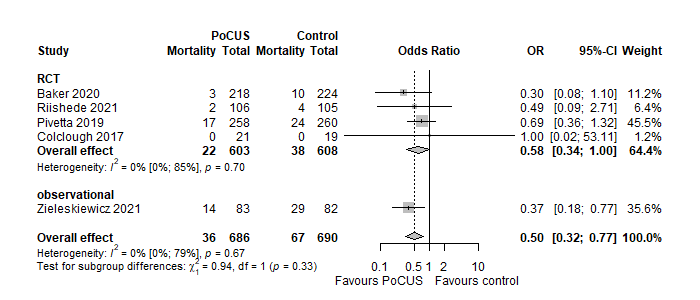
**C** Mortality – in-hospital (Laursen 2014 leaved out)


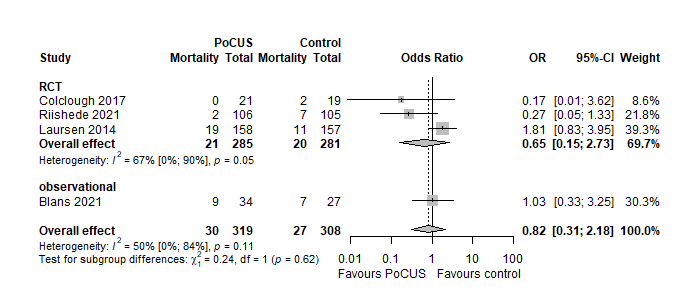
**D** Mortality – 30-day

**--------------------------------------------------------------------------------------------------------------------------------------**

Comparison of patients admitted with dyspnea examined by PoCUS vs conventional modalities in Length of stay – in-hospital (considerable heterogeneity detected) (A), Length of stay – Emergency Department (low heterogeneity detected) (B), In-hospital mortality with the study by Laursen 2014 leaved out (The possible explanation for the opposite direction of the result could be that it was an older single center study focusing on diagnostic accuracy as a primary outcome and all the ultrasounds were made by one person, who, although an expert, could be a cause of bias. Therefore, we performed the leave-one-out analysis, resulting in patients in the PoCUS group having a significantly lower risk of in-hospital death than controls.) (moderate heterogeneity detected) (C), and 30-day mortality (moderate heterogeneity detected) (D). PoCUS indicates Point of Care Ultrasound; SD, standard deviation; MD, mean difference. The size of squares is proportional to the weight of each study. Horizontal lines indicate the 95% CI of each study; diamond, the pooled estimate with 95% CI.

**Additional Figure 2 – Funnel Plots of the Meta-Analysis**

Time to diagnosis


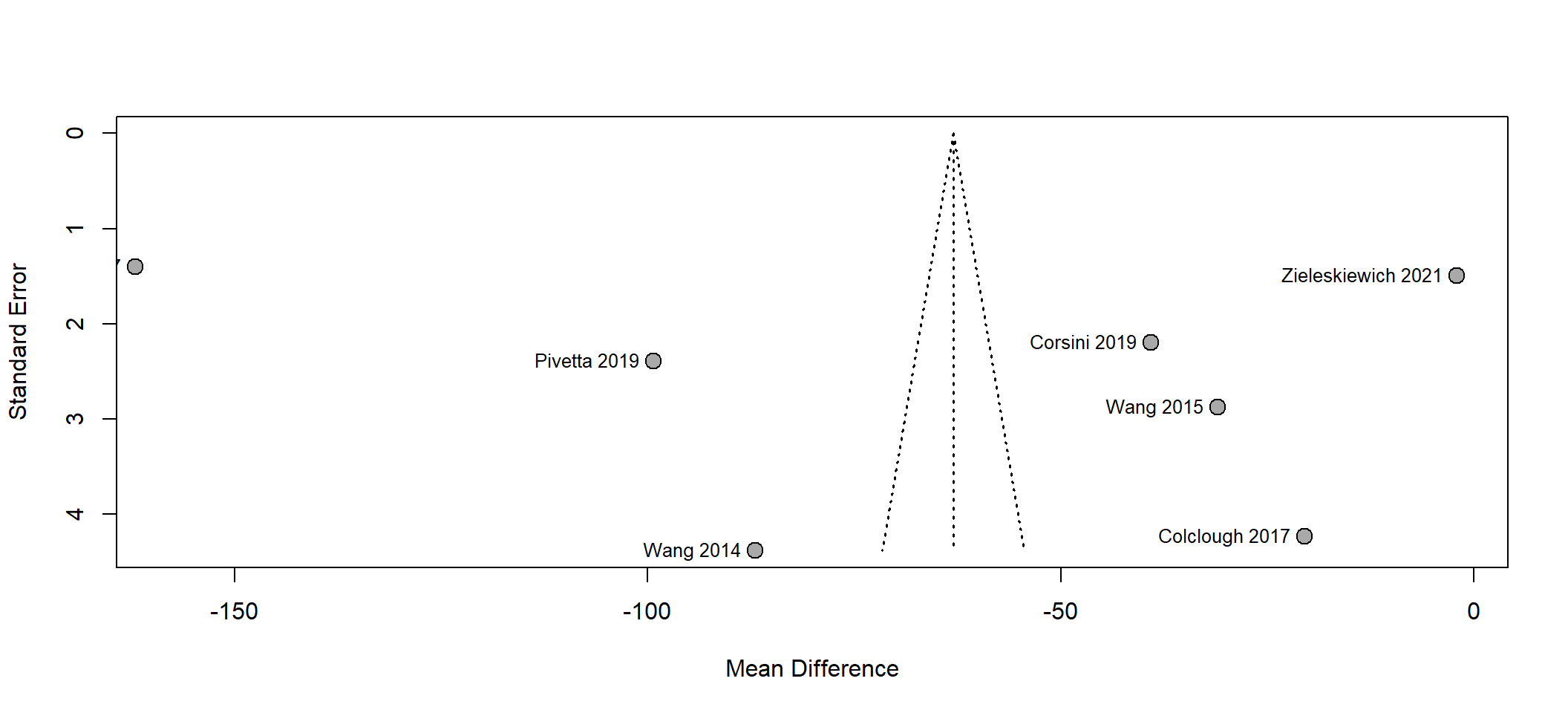


Time to treatment


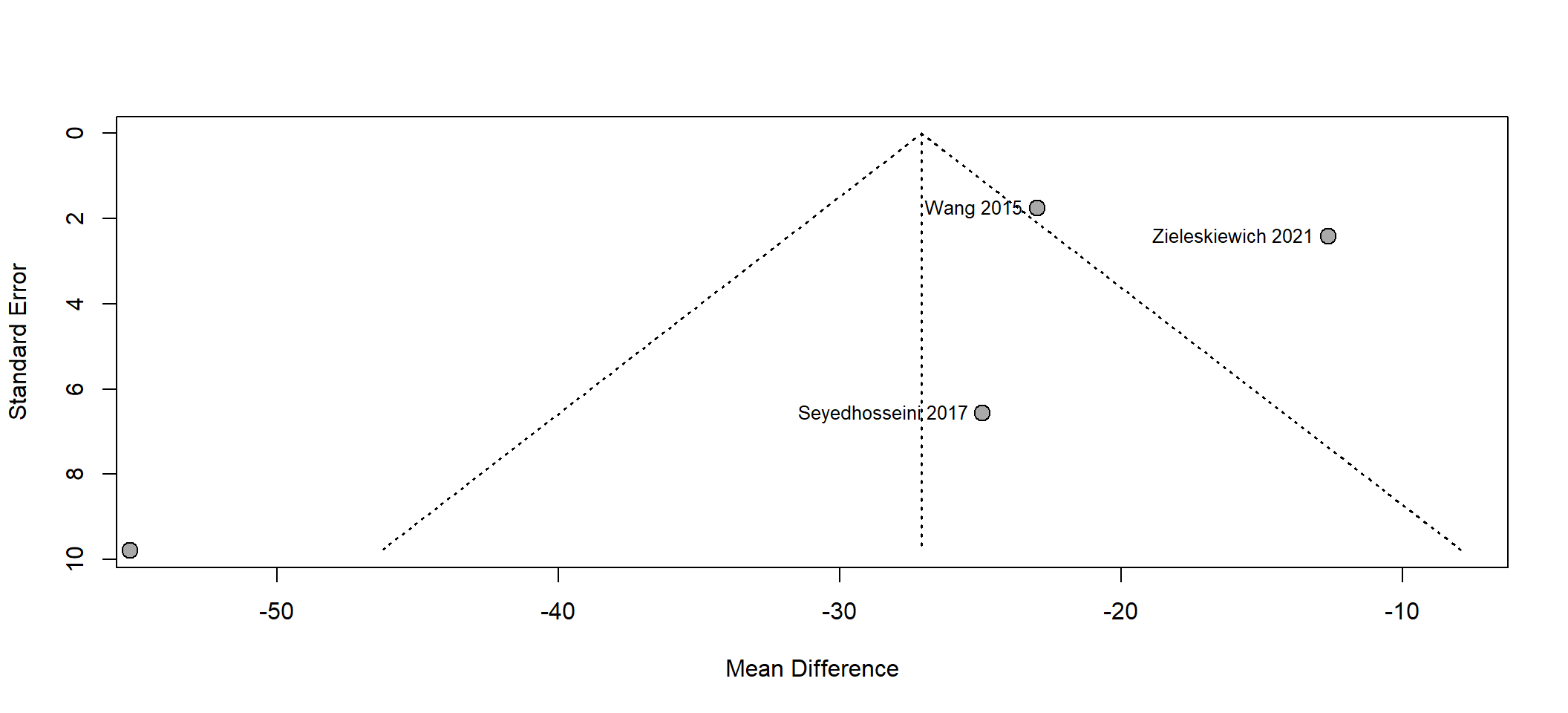


Length of stay – inhospital
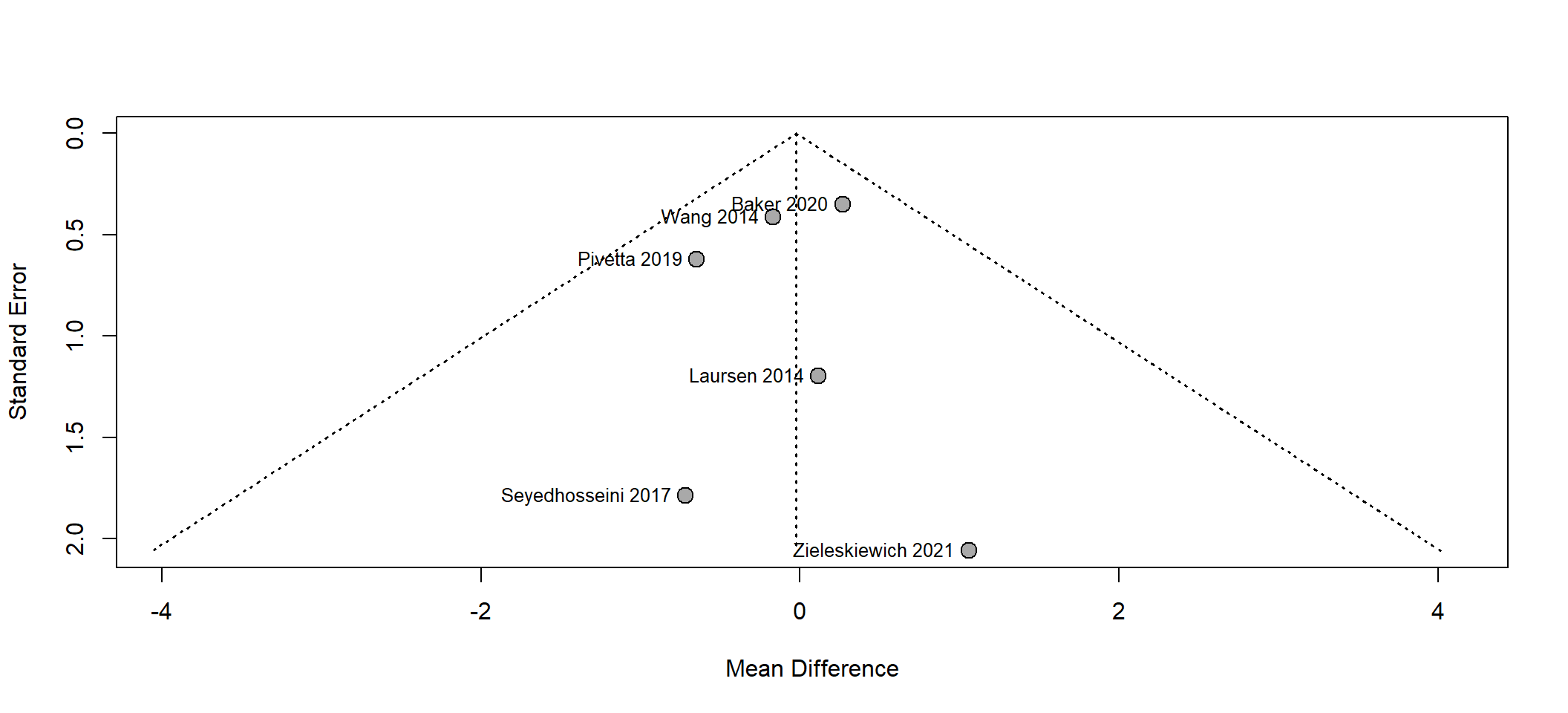


Length of stay – Intensive Care Unit


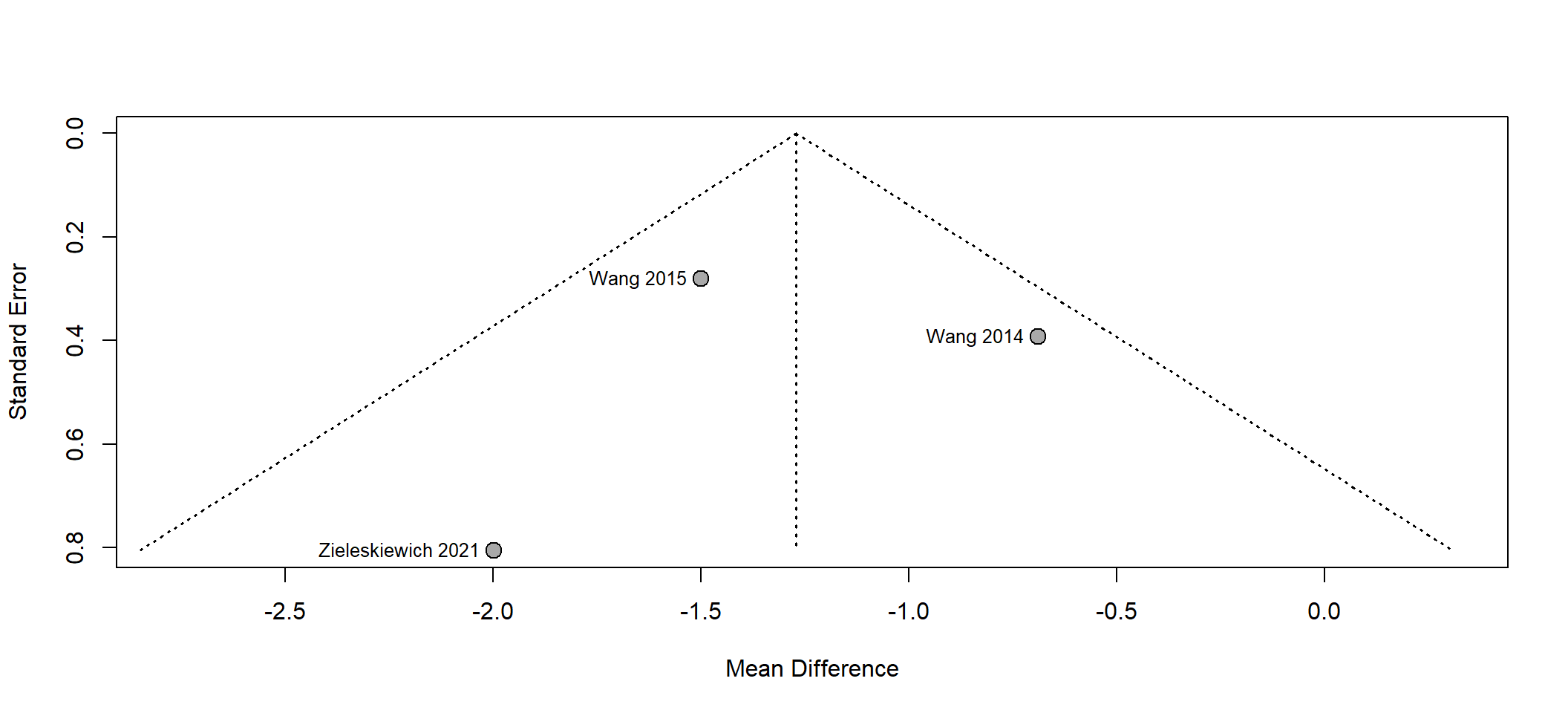


Length of stay – Emergency Department


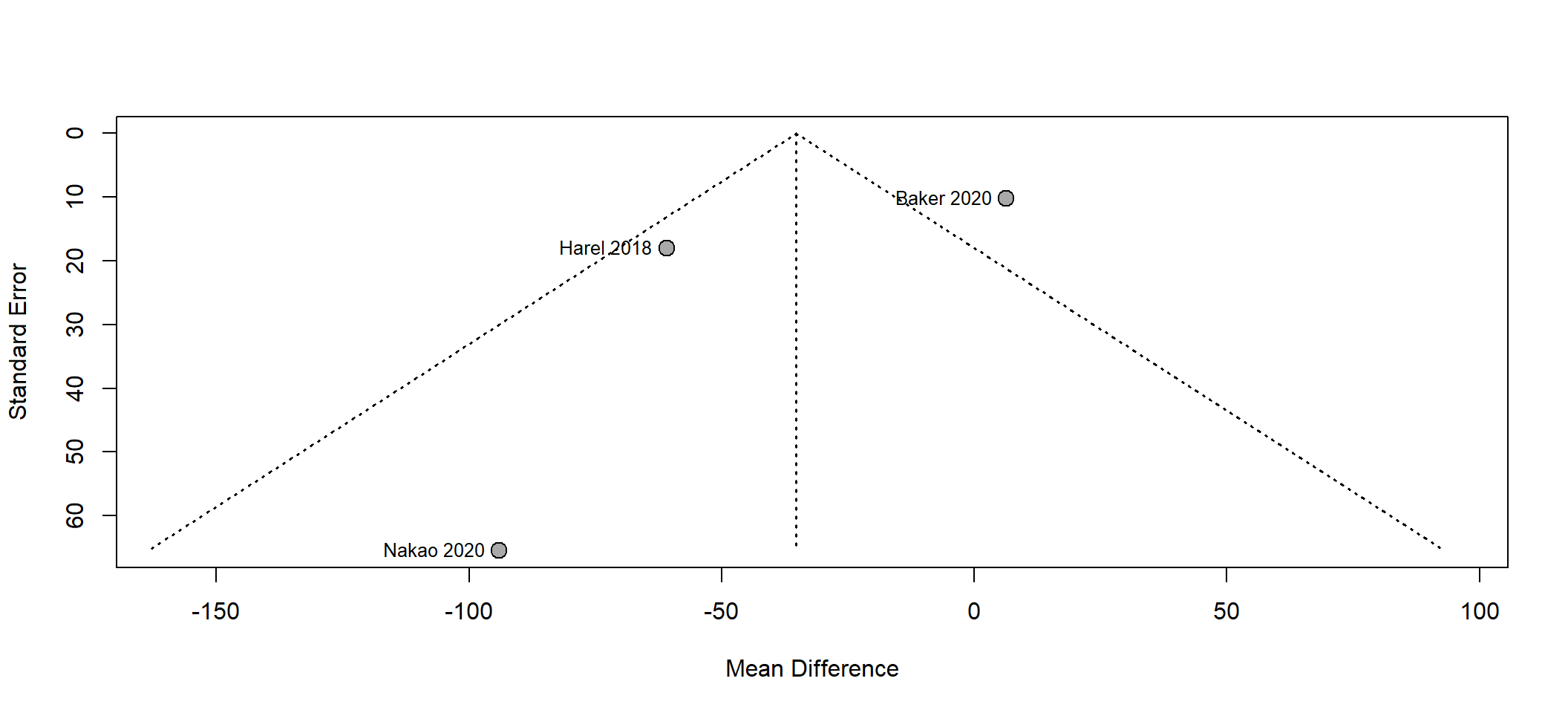


Rate of appropriate treatment


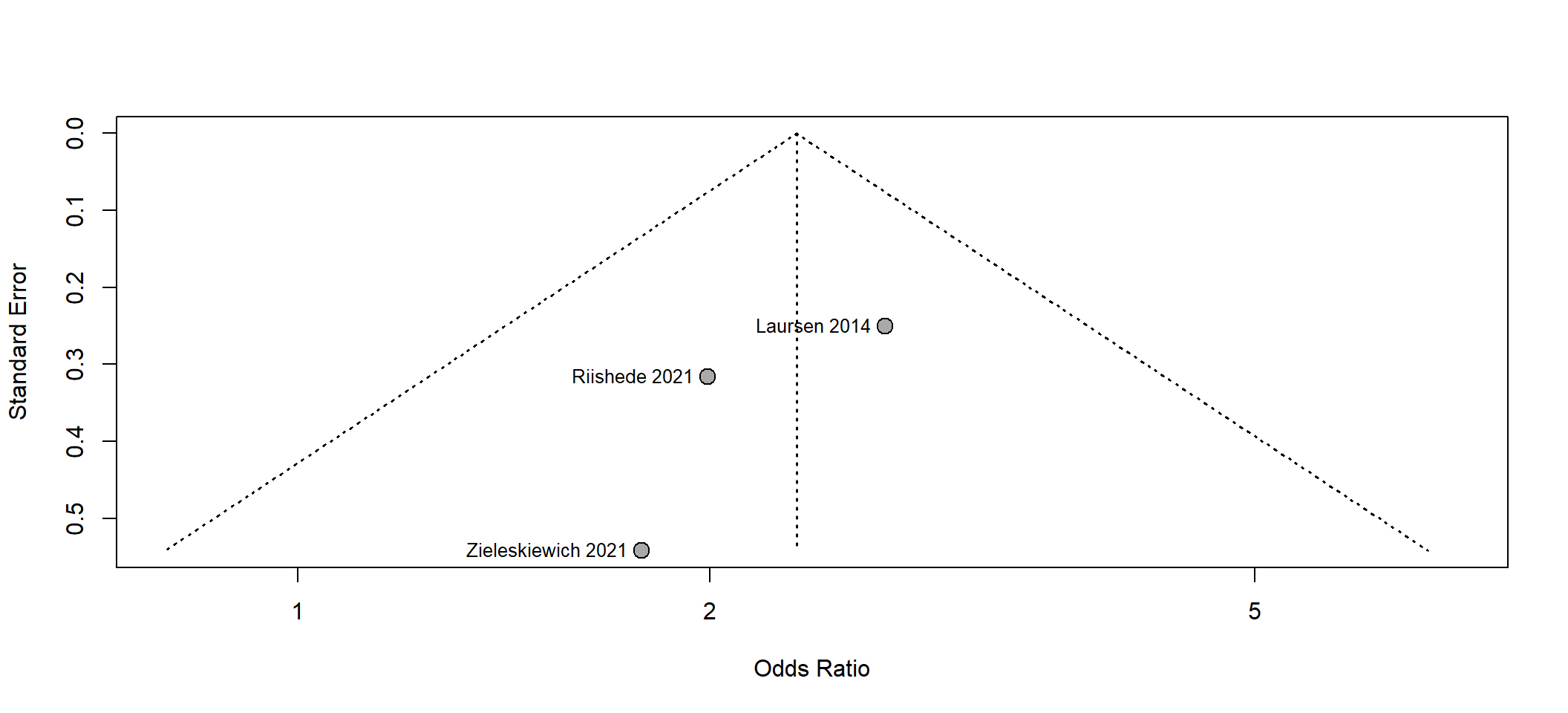


30-day readmission rate


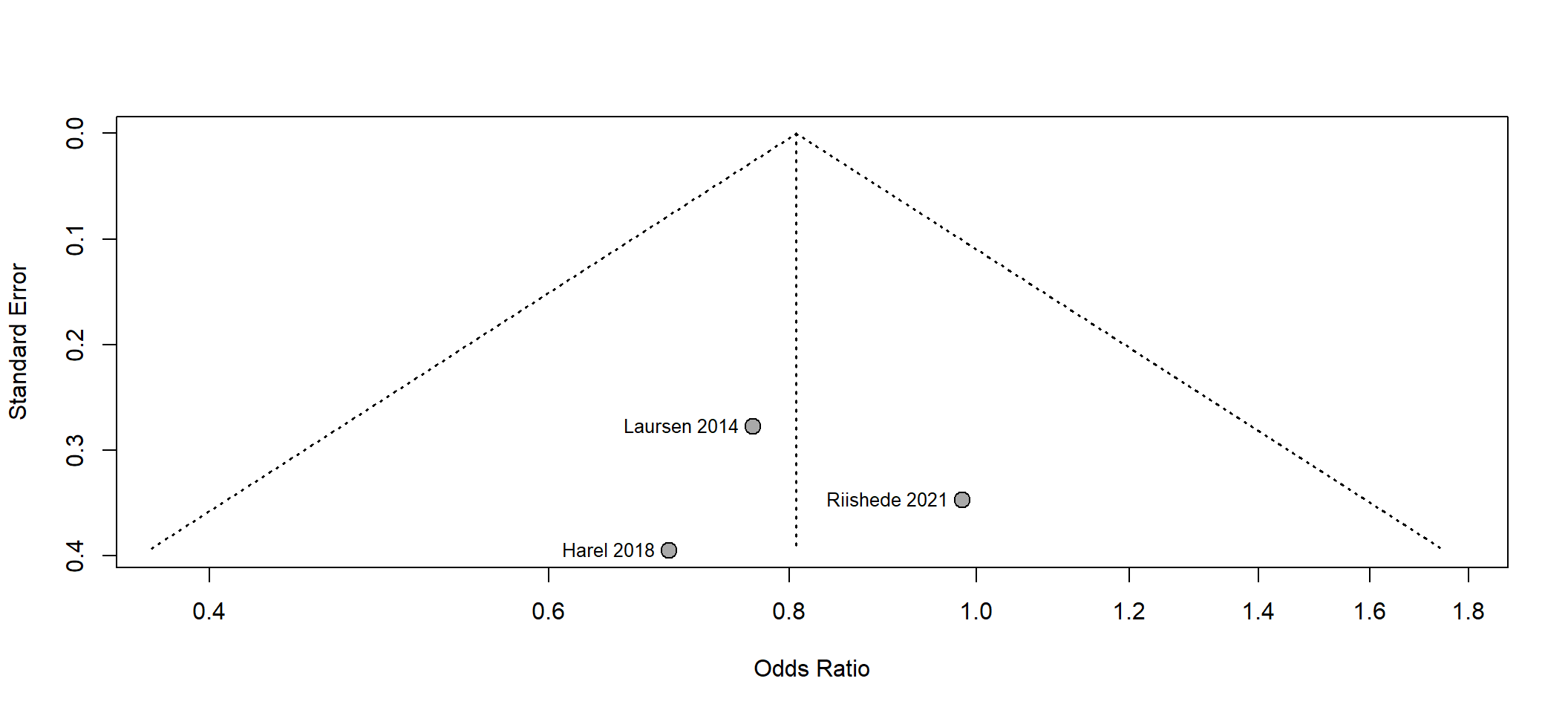


Mortality – 30-day
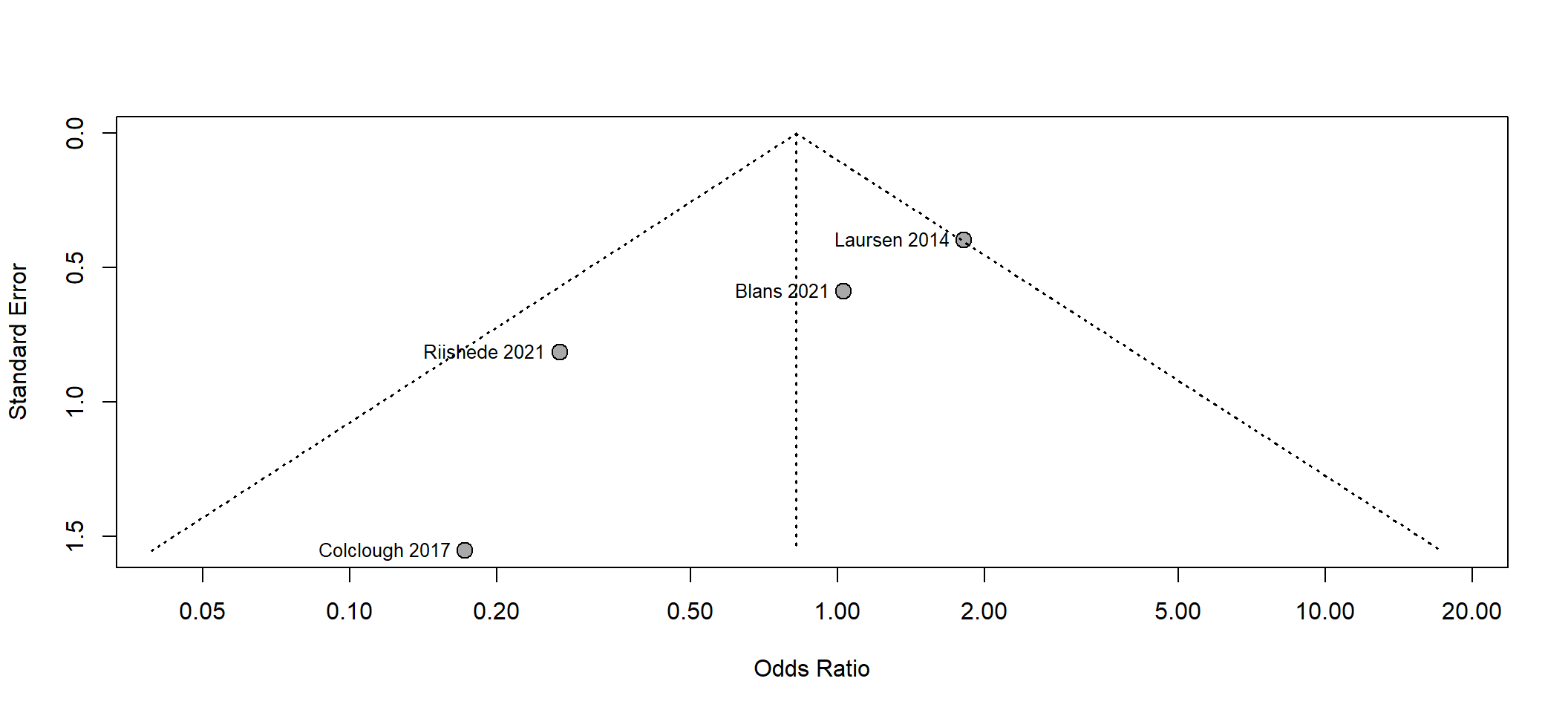


Mortality – in-hospital


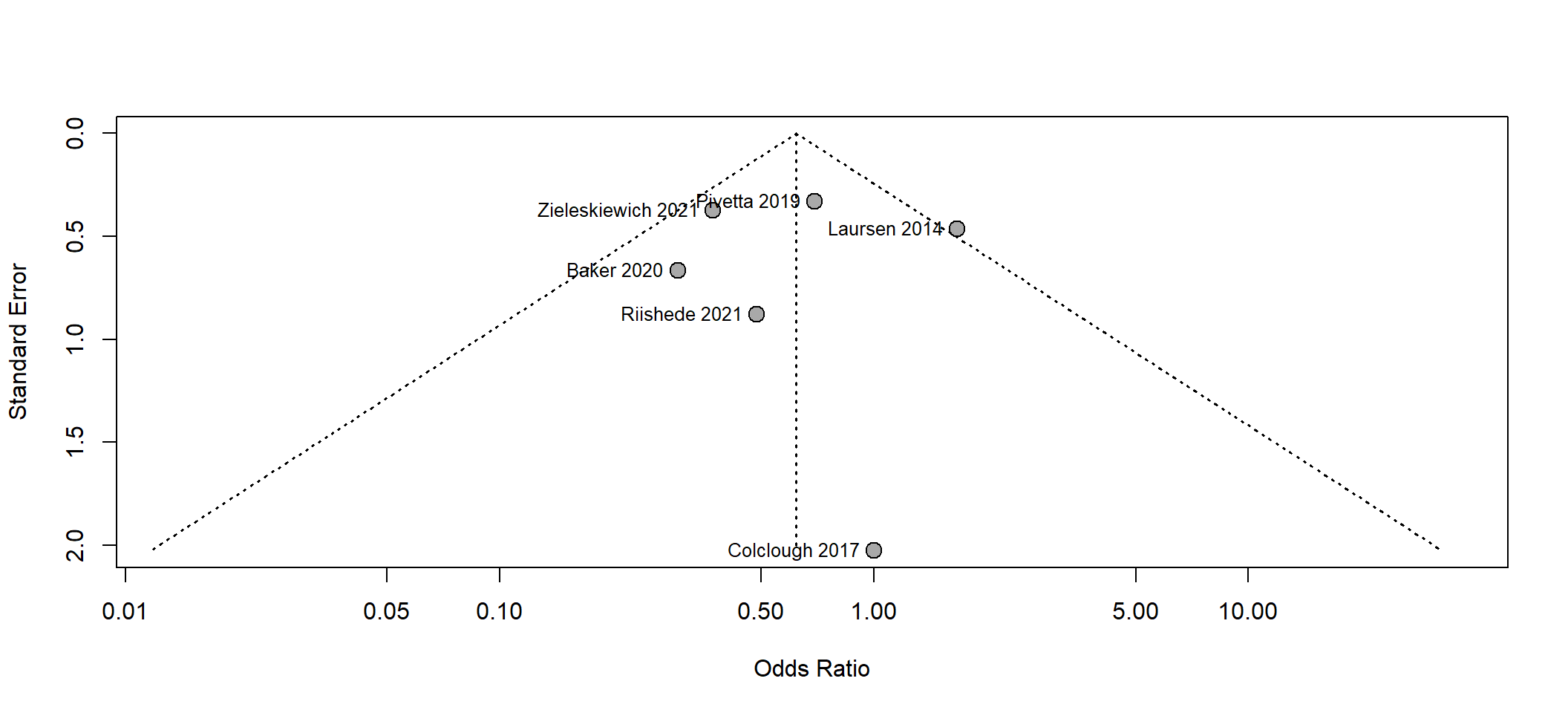
Mortality – in-hospital (without Laursen 2014)


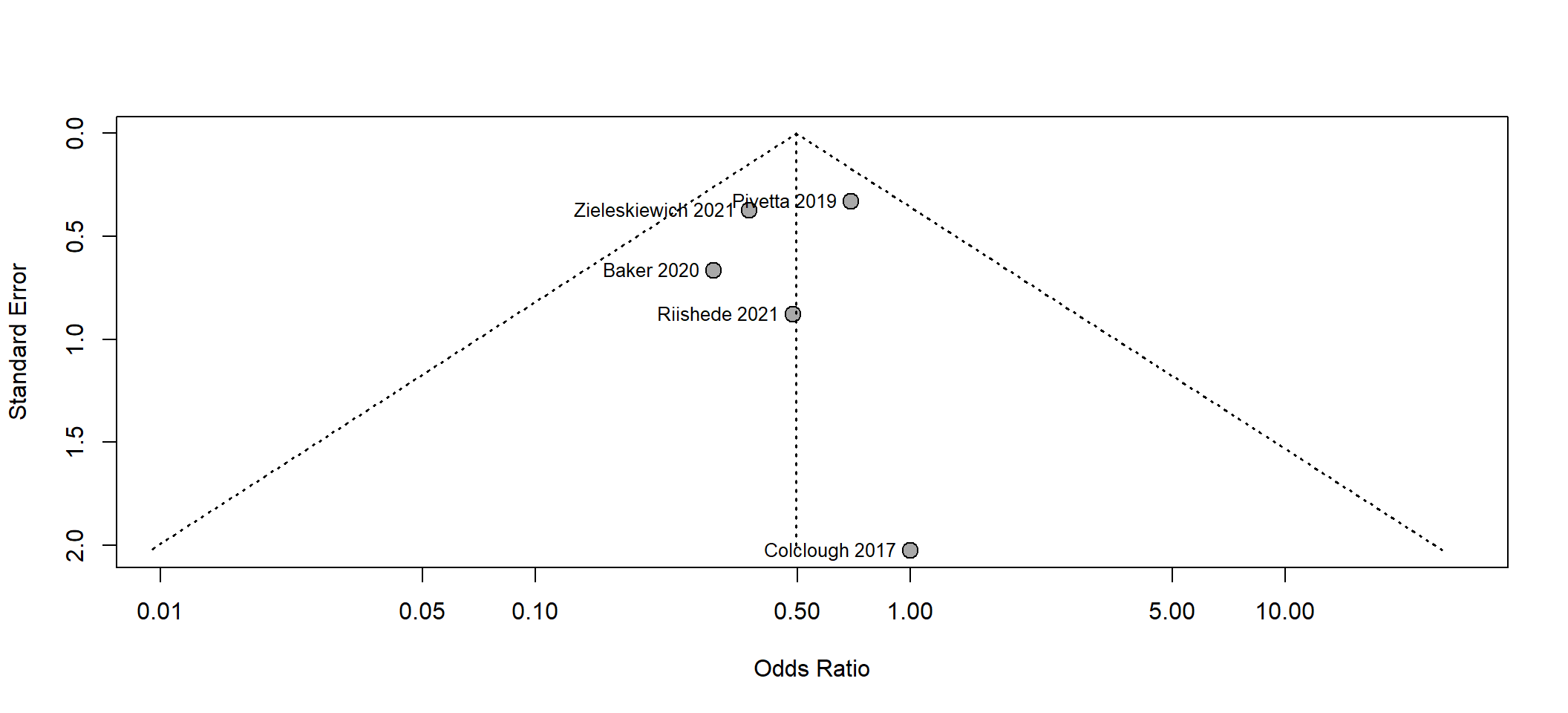


Based on the visual inspection of the Funnel plots no small study effect was identified. The study number is limited (<10) for each outcome so the results should be handled with high care.

**Additional Figure 3 – The leave-one-out sensitivity analyses**

Length of stay


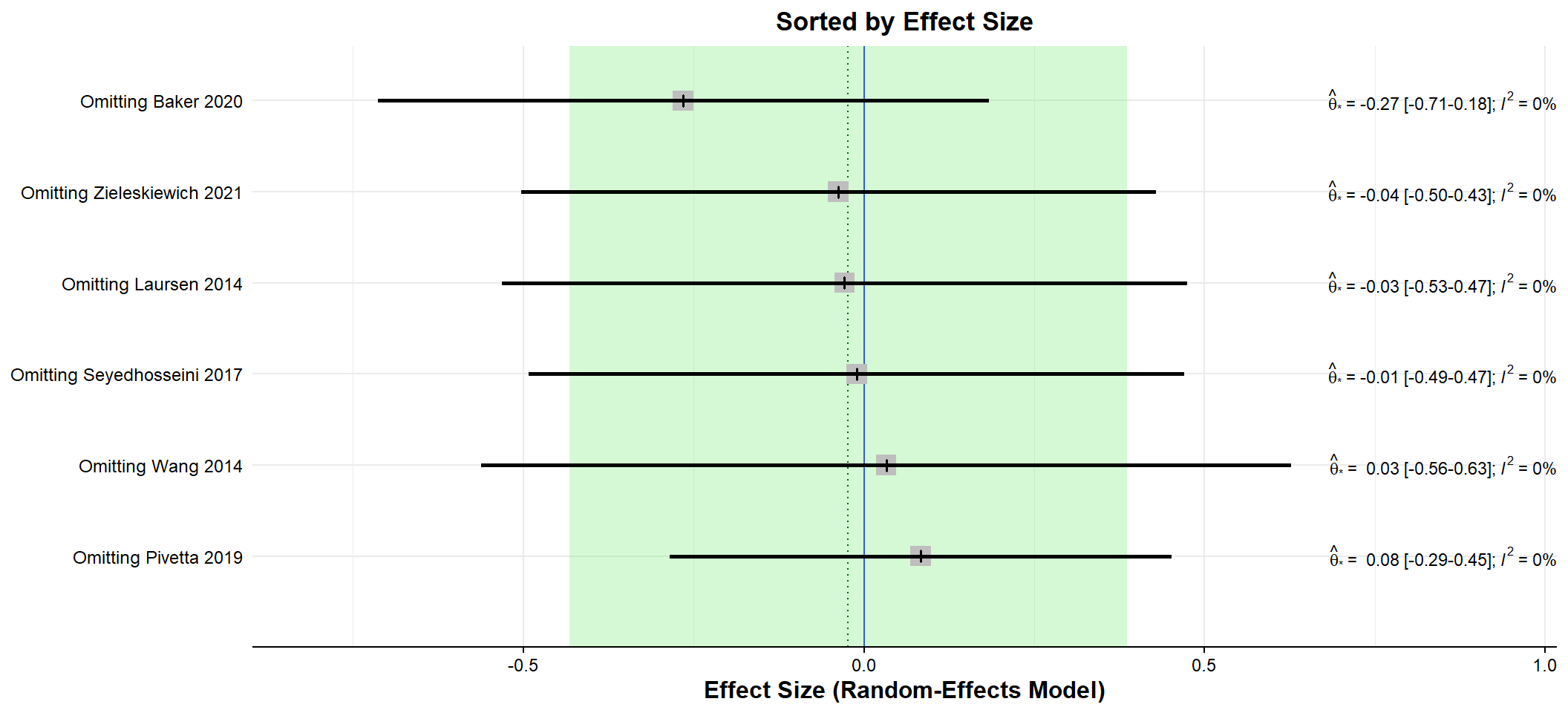


Mortality – in-hospital


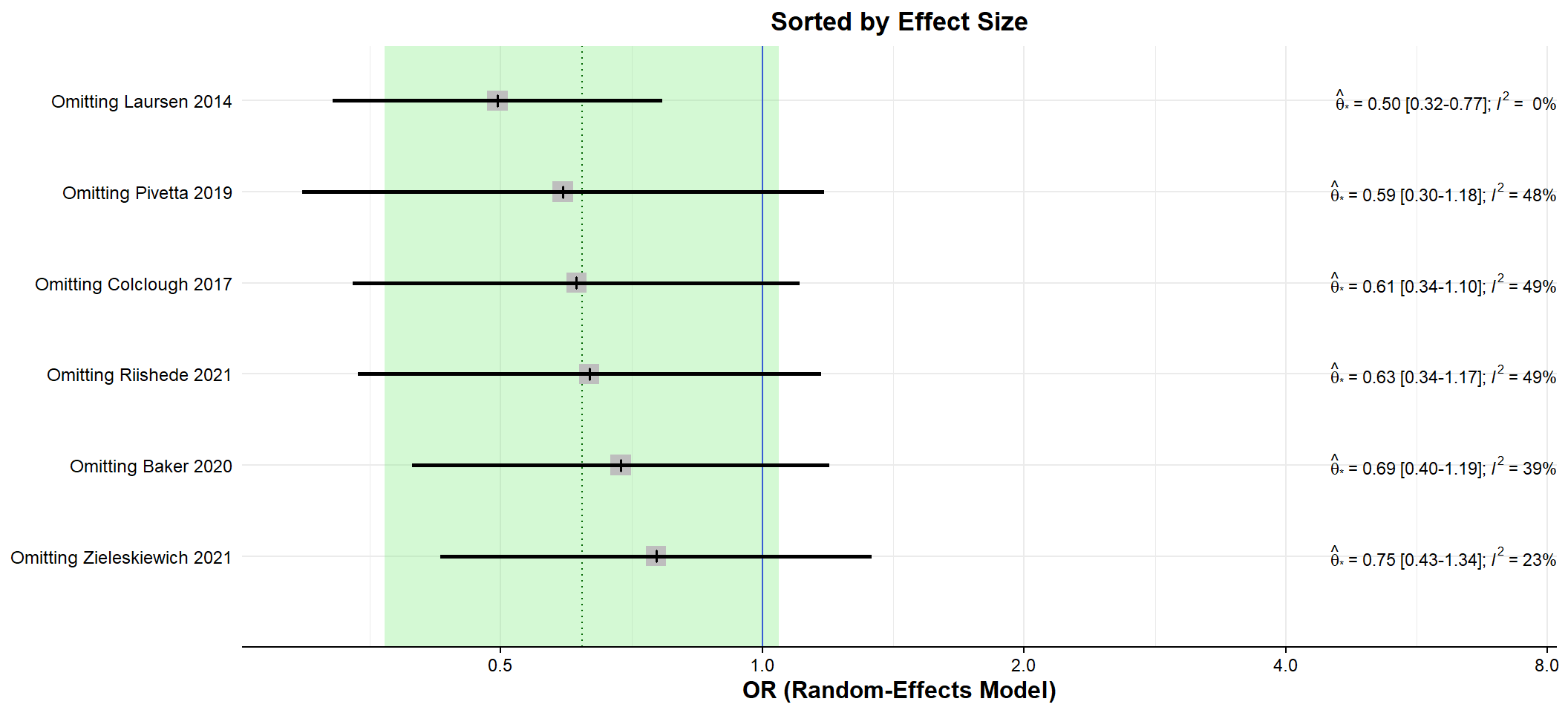


Time to diagnosis


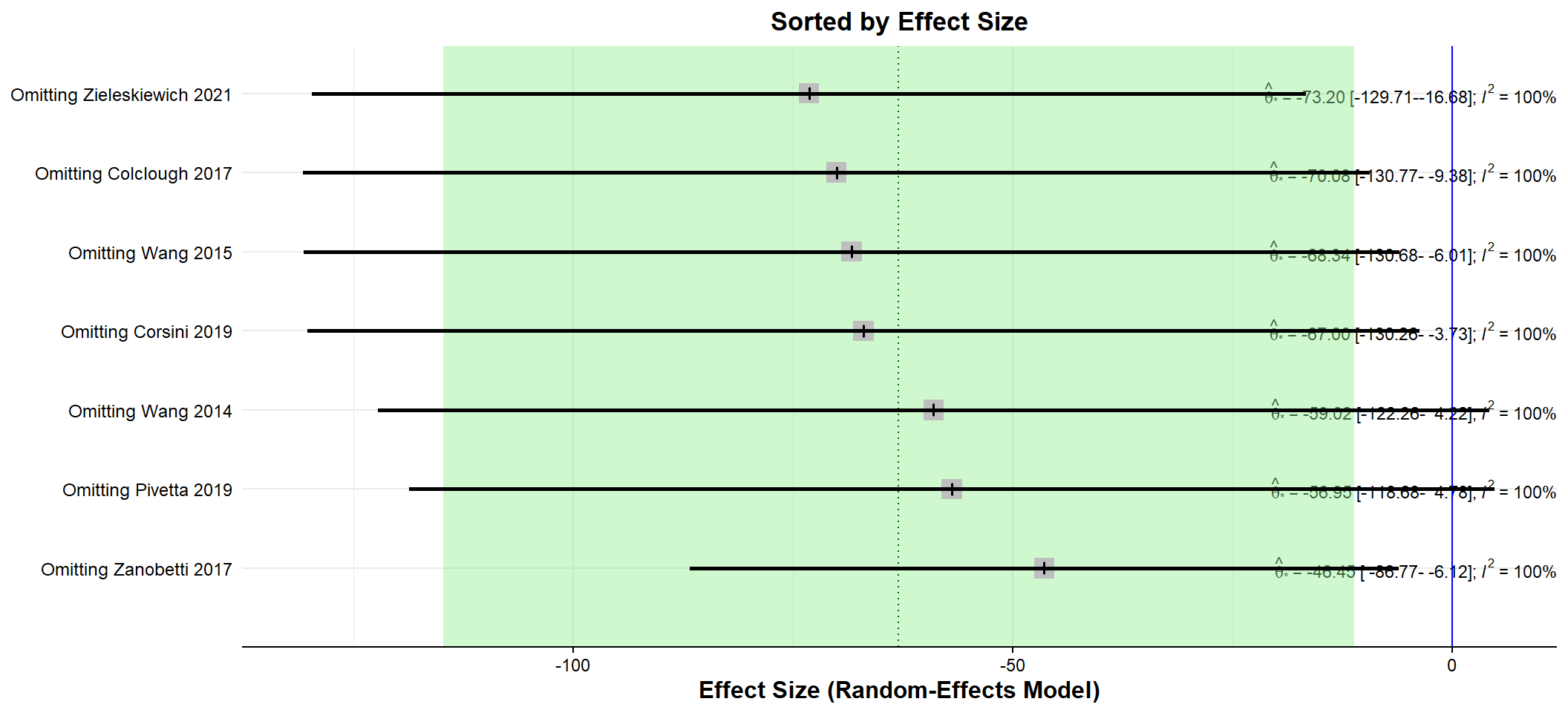


Results of leave-one-out sensitivity analysis (plot and numbers) in studies. The vertical axis represents the omitted study. The horizontal axis shows the odds ratio (OR) or effect size. The shaded area with a dashed line in its center represents the 95% confidence interval of the original pooled effect size and the estimated pooled effect itself. In the right part of the figure the new heterogeneity (*I^2^*) number is visible after omiting the evaluated study.

This leave-one-out sensitivity analysis only be perfomed if at least five studies available for the same outcome. Omitting Laursen 2014 results only any meaningful change in the heterogeneity (*I^2^*), for further details see the comment of Additional Figure 1

**Additional Method: Search key**

EMBASE:

('point of care'/exp OR 'point of care' OR portable OR bedside OR 'bed side' OR handheld OR 'hand held' OR 'hand carried' OR pocket OR mobile) AND (ultrasoun* OR ultrason* OR sonogr* OR echo*) AND (pneumo* OR bronchopneumon* OR pleuropneumon* OR chylothora* OR hemothora* OR haemothora* OR hydropneumothora* OR hydrothora* OR ((pulmo* OR 'lung'/exp OR lung OR 'vein'/exp OR vein) AND (edem* OR oedem* OR 'congestion'/exp OR congestion OR embol* OR thromb*)) OR (('heart'/exp OR heart OR cardiac OR circula* OR resp*) AND ('failure'/exp OR failure OR 'distress'/exp OR distress OR insufficien*)) OR dyspn* OR breathless* OR (short* AND of AND ('breath'/exp OR breath)))

Pubmed and CENTRAL:

(("point of care" OR point-of-care OR portable OR bedside OR bed-side OR handheld OR hand-held OR hand-carried OR pocket OR mobile) AND (ultrasoun* OR ultrason* OR sonogr* OR echo*)) AND (pneumo* OR bronchopneumon* OR pleuropneumon* OR chylothora* OR hemothora* OR haemothora* OR hydropneumothora* OR hydrothora* OR ((pulmo* OR lung OR vein) AND (edem* OR oedem* OR congestion OR embol* OR thromb*)) OR ((heart OR cardiac OR circula* OR resp*) AND (failure OR distress OR insufficien*)) OR dyspn* OR breathless* OR (short* of breath))
